# The potential of eHealth for cancer patients–does COVID-19 pandemic change the attitude towards use of telemedicine services?

**DOI:** 10.1371/journal.pone.0280723

**Published:** 2023-02-10

**Authors:** Tobias A. W. Holderried, Katharina Hecker, Laura Reh, Martin Kirschner, Jeanette Walter, Peter Brossart, Martin Holderried

**Affiliations:** 1 Department of Oncology, Hematology, Immuno-Oncology and Rheumatology, University Hospital Bonn, Bonn, Germany; 2 Center for Integrated Oncology Aachen Bonn Cologne Düsseldorf (CIO ABCD), Düsseldorf, Germany; 3 Institute of Econometrics and Statistics, University of Cologne, Cologne, Germany; 4 Department of Hematology, Oncology, Hemostaseology and Stem Cell Transplantation, RWTH Aachen University, Aachen, Germany; 5 Department of Quality Management, Medical and Business Development, University Hospital of Tübingen, Tübingen, Germany; The Education University of Hong Kong, HONG KONG

## Abstract

**Background:**

Internet penetration worldwide has increased rapidly over the recent years. With this growth, modern information and communication technologies (ICT) have become increasingly important. They do not only change daily life but also patient-physician interaction and health related information search, which can be summarized as electronic Health (eHealth). eHealth was already known before the emergence of the coronavirus disease 2019 (COVID-19), but this pandemic substantially challenged health systems, physicians and hospitals so profoundly that new services and methods of patient-physician interaction had to be implemented rapidly. This study investigates the attitude of cancer patients towards eHealth and the potential impact of COVID-19 on its use.

**Methods and findings:**

The study was a multicentered study carried out at the university hospitals Bonn and Aachen. Patients were asked to answer a structured questionnaire in the time span between September 2019 and February 2021. Due to the COVID-19 pandemic, no patients were addressed between March 2020 and July 2020. The questionnaire focused on socio-demographic data, the dissemination of internet-enabled devices, the patients’ attitude towards eHealth and the use of modern ICT in daily life and for health-related information search. In total, 280 patients have filled the questionnaire of which 48% were female and 52% were male. Men have a slightly more positive attitude towards the overall potential of eHealth than women which was shown by a significant influence for receiving medical information via e-mail. Hematological-oncological patients with a higher education level reported a significantly higher willingness to send personal health information to their physician and health insurance. A frequency of medical consultation of more than 5 times during the previous year has a significantly positive impact regarding the use of online communication, online video consultation and treatment quality. Younger patients have more concerns about data security than older patients. The study shows a different attitude towards the influence of eHealth on the patient-physician relationship in different therapy situations. While there were no significant changes in patients’ attitude towards eHealth after the start of the COVID-19 pandemic, there was a trend towards an increasingly embracing attitude in patients, who answered the questionnaire during COVID-19 pandemic situation.

**Conclusions:**

Overall, cancer patients had a positive attitude towards eHealth and the dissemination of internet-enabled devices was high. The study shows that the potential of eHealth is high among hematological-oncological patients. Further eHealth technologies and especially telemedically supported care processes should be implemented to improve patient-physician interaction and cross-sectoral care. COVID-19 pandemic led to a fast initiation and acceleration of new structures and routines for physicians, hospitals and patients. These new processes should be used to promote digitalization in hematological and oncological telemedicine. To successfully implement new eHealth technologies, future research should focus on patients’ concerns about data privacy and data availability especially in the context of exchange of medical information in cross sectoral and interdisciplinary care processes.

## Introduction

In March 2021, 65,6% of the world’s population were using the internet. Internet penetration in Europe was 88,2% and in Germany it was at 96%, which corresponds to 79.127.551 people (31.12.2020). Five years earlier (June 2016), 49,5% of the worldwide population were using the internet and in March 2011 only 30,2%. These numbers show the rapid increase of internet penetration and use over the last ten years [1]. Modern information and communication technologies (ICT) become increasingly important not only in daily life, but also for health-related information search and patient-physician interaction, which can be summarized as electronic Health (eHealth) [2–5].

The WHO summarizes eHealth as follows: “e-Health is the cost‐effective and secure use of information and communication technologies in support of health and health‐related fields. It encompasses multiple interventions, including telehealth, telemedicine, mobile health, electronic medical or health records, big data, wearables, and even artificial intelligence. The role of eHealth has been recognized as pivotal in attaining overarching health priorities such as universal health coverage and the Sustainable Development Goals” [6]. A subcategory of eHealth is teleoncology. Teleoncology describes the use of modern ICT for communication between physicians, hospitals and cancer patients. Teleoncology can be used for both patients receiving active therapy and follow-up patients [7]. Increasing importance of modern ICT has led to a change in the healthcare system and patient-physician communication [4, 8]. There has been an increase in internet-based queries for health topics [9–13]. A study conducted in Poland showed that health related online information search increased from 41,7% in 2005 to 66,7% in 2012 [14]. A couple of years later, Dutch cancer patients were asked about their health related and cancer related internet use. In this study, among the patients who use the internet in general, 63% indicated that they also use the internet for health-related information search. 49% of the general internet users responded that they use the internet for cancer related information search. General internet use showed a significance with age and education level, cancer related internet use showed significance with age. A higher fraction of younger patients was using the internet for cancer related topics than older patients [15].

The Coronavirus disease 2019 (COVID-19) pandemic is highly challenging for oncological patients due to their increased vulnerability and it is equally so for physicians, nurses, hospitals and care givers. Most healthcare providers were urged to rapidly implement services such as online chats or video consultations, especially for outpatient services. Implementing new eHealth strategies following COVID-19 might change future patient-physician interaction substantially. Teleoncology, especially online communication, will have a high impact on our daily life long after COVID-19 [7, 16–19]. The European Society of Medical Oncology guidelines for cancer patient management during COVID-19 suggest the implementation of telemedicine services for patients receiving active treatment [20]. A study conducted in Israel in 2020 showed that most patients (84,9%) wished to continue telemedicine services which had been implemented very fast to ensure cancer patients’ quality of therapy during COVID-19, also after the pandemic. Patients who were in follow up care were more likely to continue telemedicine use than patients with active oncological treatment [18].

Even with the unquestionable potential of eHealth for cross sectoral care of cancer patients, only little is known about the patients’ attitude towards the use of modern information and communication technologies and whether the attitude has changed during COVID-19 pandemic situation [18, 21]. The objective of the present study was to investigate the attitude of cancer patients towards eHealth and how use of eHealth changed during the COVID-19 pandemic. To this end, patients were asked to assess their use of modern ICT for daily life and health-related information search and rate their attitude towards eHealth and use of modern online communication with their physicians or hospitals.

## Methods

### Design

The multicenter study was a prospective, questionnaire-based study. Structured questionnaires were addressed to patients in the time span September 2019 –February 2021. Due to the unclear situation at the beginning of the COVID-19 pandemic, no patients were addressed between March 2020 and July 2020.

### Ethics

This survey-based study was approved by the IRB (Institutional Ethics Committee of the Medical Faculty and University Hospital of Bonn, approval number 386/19). Oral consent was obtained from all participants and confirmed by the participants by return of the completed survey.

### Patients

In total, 305 patients that underwent outpatient chemotherapy treatment or post-chemotherapy after care were asked to participate in the survey-based study and 280 patients filled the questionnaire. All patients were contacted personally at the University Hospital Bonn, Department of Internal Medicine, Hematology and Oncology and the University Hospital Aachen, Department of Hematology, Oncology, Hemostaseology and Stem Cell Transplantation. Inclusion criteria were underlying malignant disease currently undergoing outpatient chemotherapy treatment or after-care. Each patient was ≥ 18 years old. For statistical analysis, the patients were divided into two age groups ≤ 54 and ≥ 55 years old, motivated by the significant increase in the risk of developing cancer from the age of 55 in Germany [22].

### Questionnaire

**Fig 1:**
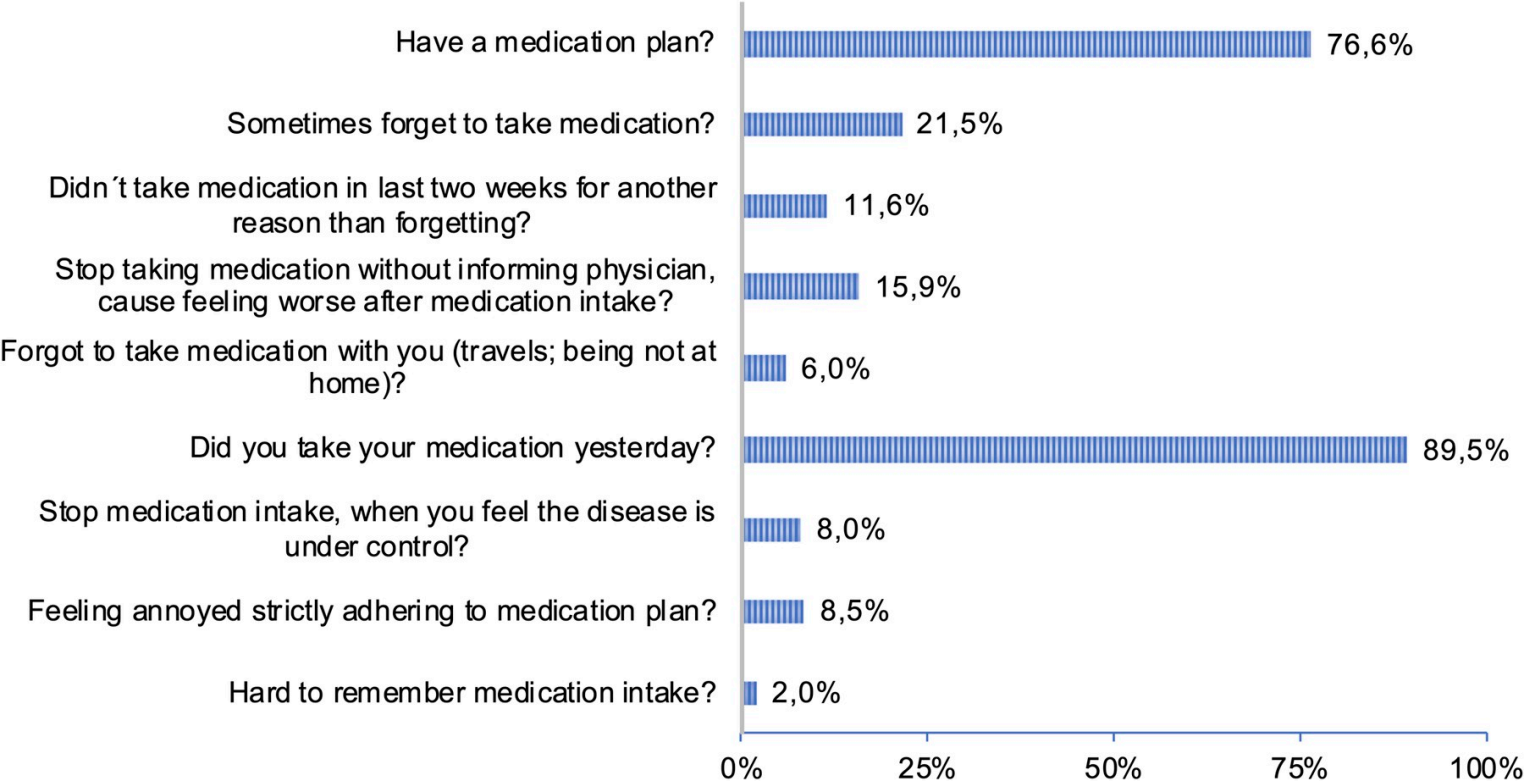
Medication plan and medication intake.

**Fig 2:**
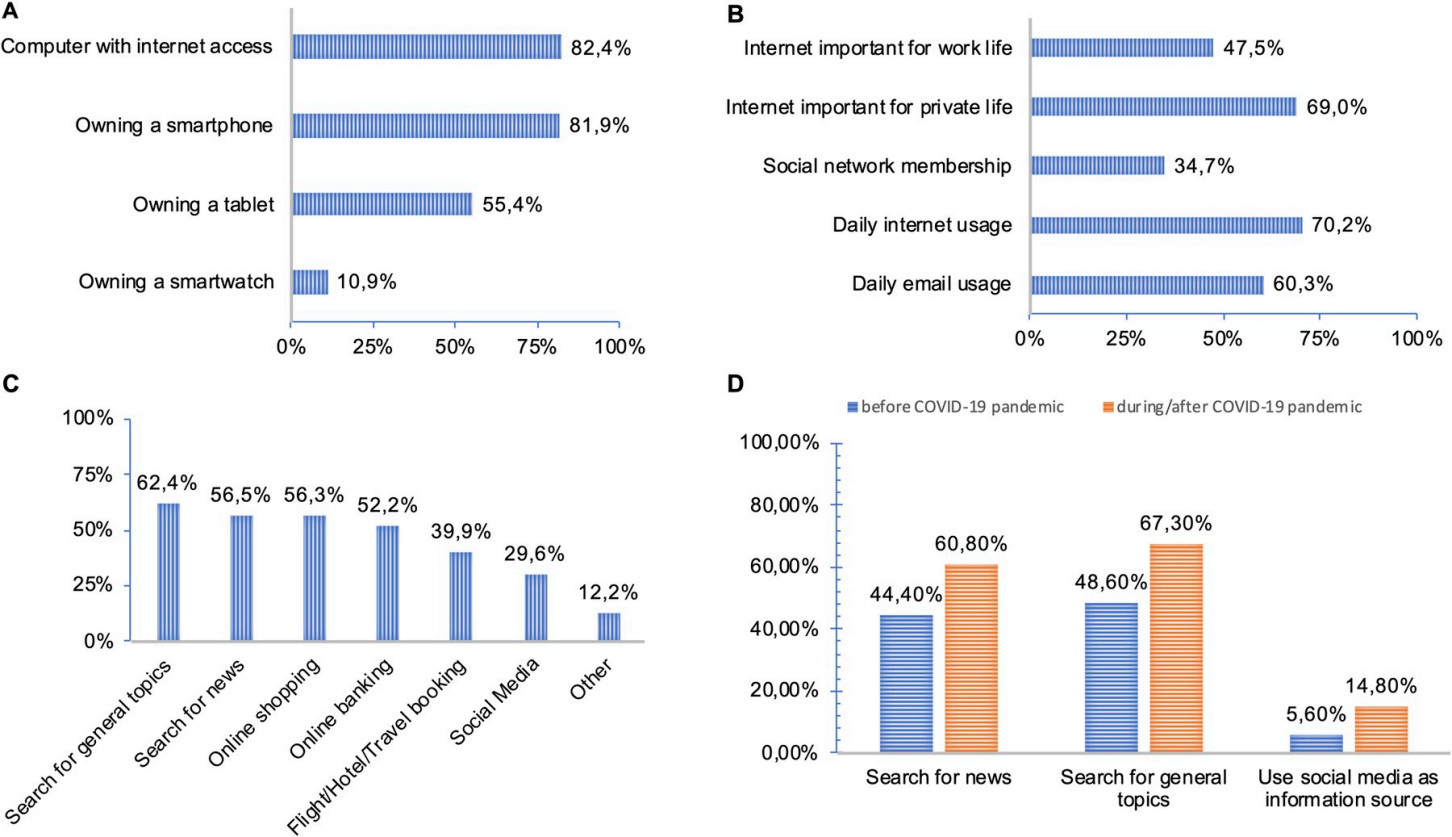
A. Dissemination of internet-enabled devices. B. Penetration and use of modern ICT in daily life. C. Non-health-related topics cancer patients search for online. D. Usage of Online Services and COVID-19 pandemic.

**Fig 3:**
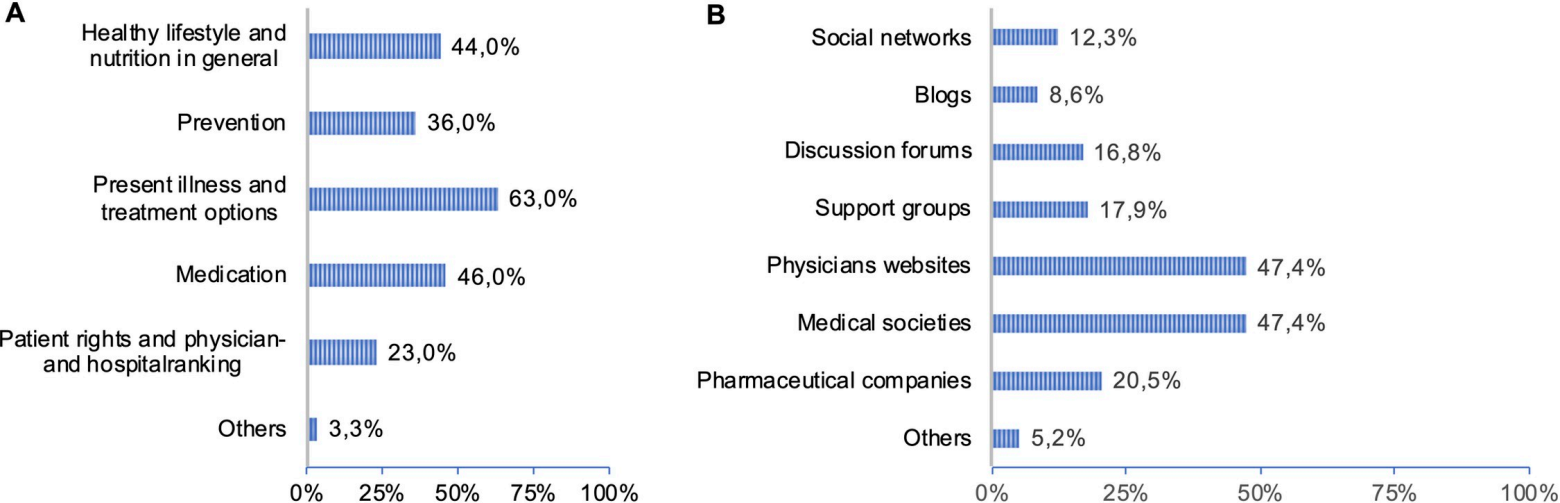
A. Online health-related information search. B. Sources of online health information.

**Fig 4:**
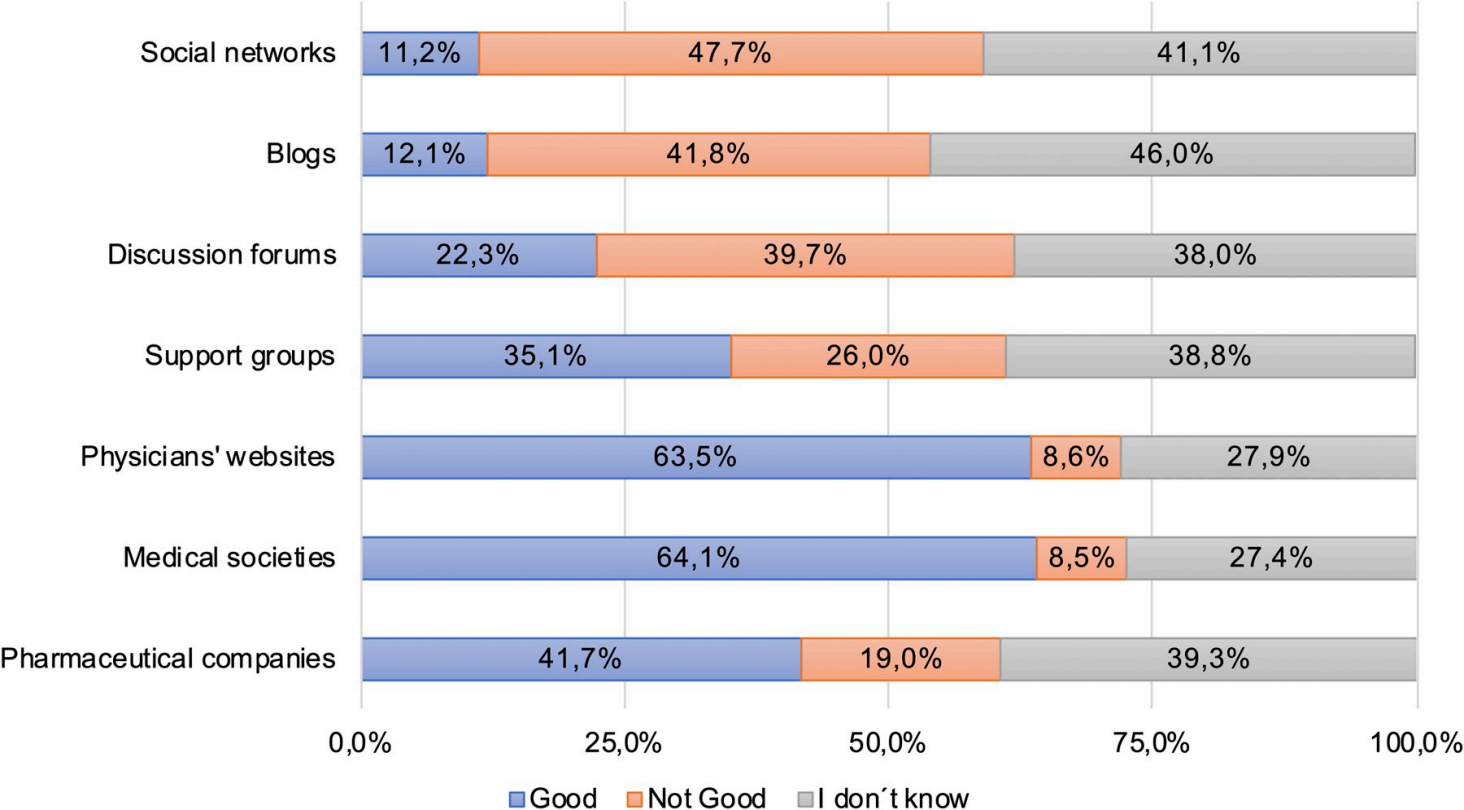
Patients’ assessment of the quality of online sources for health-related information.

**Fig 5:**
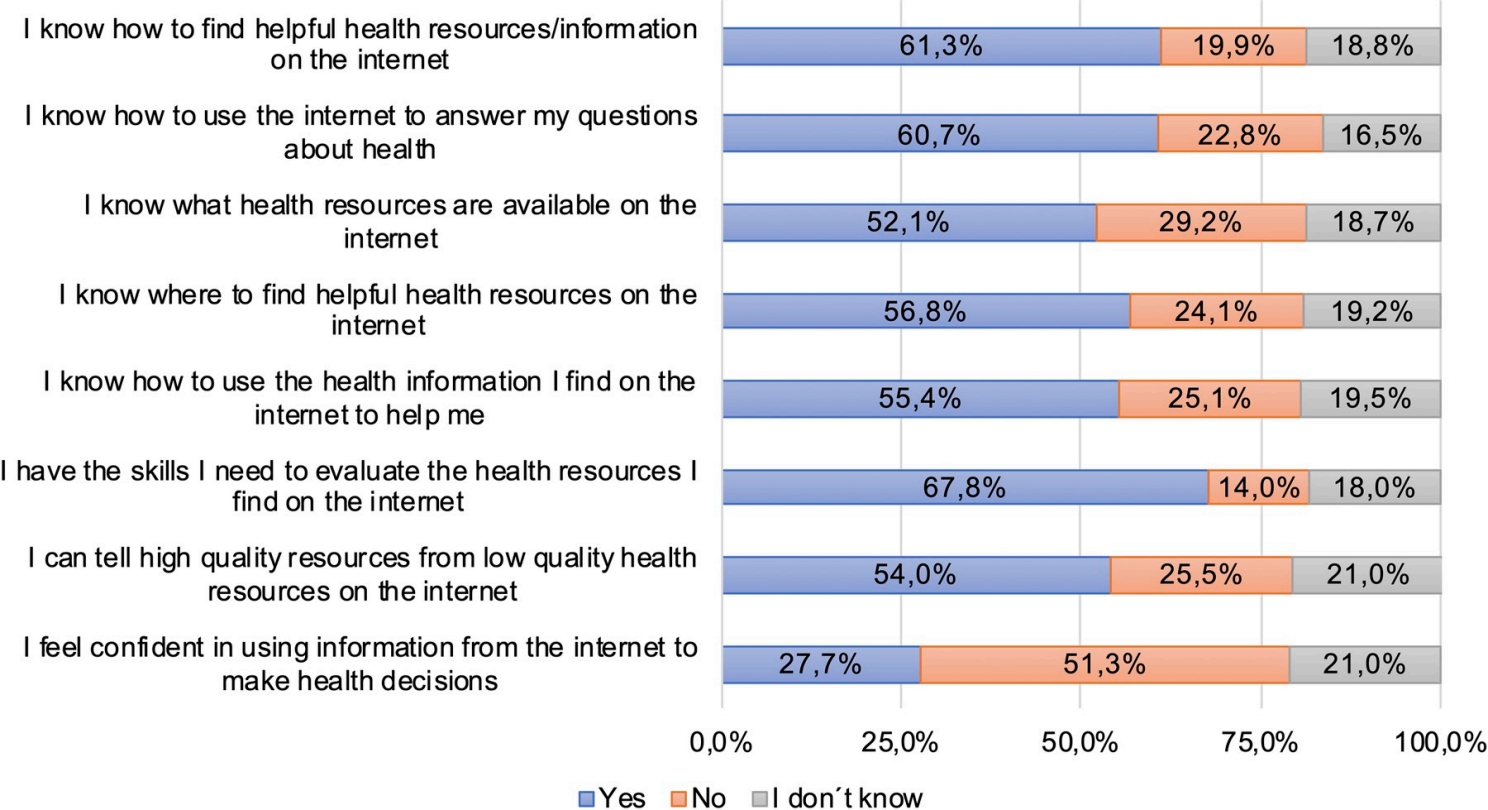
Results of the eHealth Literacy Scale (eHEALS).

The structured, paper-based questionnaire was developed by an interdisciplinary team of physicians specialized in hematology and oncology, eHealth specialists, quality managers and public health researchers based on the current literature and their own experience in other medical fields [21, 23–27]. The questionnaire was composed of the following study specific variables: socio-demographic data (e.g. age, education level); the current use of modern information and communication technologies in private life (e.g. smartphone possession, daily e-mail usage and online information search); online health related information search and the attitudes towards the use of eHealth for intersectoral patient-physician communication (e.g. willingness to use online health messaging or video consultations). For the questionnaire close-ended questions (e.g., smartphone possession, regular medication intake) and evaluations scales for specific measures (e.g., online communication of general and personal health-related information) were used. The surveyed aspects, including the number of statements and scale scores for each response, are summarized in detail in Tables 1 and 2 and Figs 1–5 and S1–S9 Tables. The 8-item based eHealth Literacy Scale (eHEALS) was included in the questionnaire for evaluating the perceived knowledge and skills for using digital information technology for health reasons among the study population [28–31].

**Table 1:** Age-related characteristics of the study sample (N = 280).

	Total	Age		
		≤ 54 n (%)	≥ 55 n (%)	
	n (%)			*p*-Value
**Gender**				
Female	130 (48,0)	37 (28,5)	93 (71,5)	
Male	141 (52,0)	30 (21,7)	108 (78,3)	ns. (0,204)
**Age**				
≤ 54	67 (25,0)			
≥ 54	201 (75,0)			
**Community size (inhabitants)**				
≤ 30.000	138 (52,7)	32 (23,2)	106 (76,8)	
> 30.000	124 (47,3)	32 (26,2)	90 (73,8)	ns. (0,570)
**Proximity to university hospital**				
≤ 20 km	134 (50,0)	40 (30,3)	92 (69,7)	
≥ 21 km	134 (50,0)	27 (20,3)	106 (79,7)	ns. (0,061)
**Travel time to university hospital**				
≤ 30 min	142 (53,4)	46 (32,9)	94 (67,1)	
≥ 31 min	124 (46,6)	21 (16,9)	103 (83,1)	**0,003**
**Educational level**				
Low	84 (31,7)	7 (8,4)	76 (91,6)	
Middle + high	181 (68,3)	60 (33,5)	119 (66,5)	**<0,001**
**Occupational education**				
Low	26 (9,8)	5 (19,2)	21 (80,8)	
Middle + high	238 (90,2)	62 (26,4)	173 (73,6)	ns. (0,428)
**Employed**				
No	199 (74,8)	37 (18,8)	160 (81,2)	
Yes	67 (25,2)	30 (45,5)	36 (54,5)	**< 0,001**
**Full time or part time job**				
≤ 50%	25 (36,2)	13 (52,0)	12 (48,0)	
> 50%	44 (63,8)	16 (37,2)	27 (62,8)	ns. (0,234)
**Frequency of medical consultation**				
≤ 5 times/last year	45 (17,0)	7 (15,9)	37 (84,1)	
> 5 times/last year	220 (83,0)	60 (27,6)	157 (72,4)	ns. (0,104)
**Missed appointments in the past**				
No	242 (90,0)	54 (22,9)	182 (77,1)	
Yes	27 (10,0)	13 (48,1)	14 (51,9)	**0,004**
**Insurance status**				
Statutory health insurance	187 (69,3)	47 (25,4)	138 (74,6)	
Private health insurance	83 (30,7)	19 (23,2)	63 (76,8)	ns. (0,696)
**Knowledge definition of eHealth**				
No	202 (75,1)	44 (22,3)	153 (77,7)	
Yes	67 (24,9)	23 (34,8)	43 (65,2)	**0,043**
**Medication intake**				
≤ 5 different medication/day	169 (62,8)	48 (28,9)	118 (71,1)	
≥ 6 different medication/day	100 (37,2)	18 (18,6)	79 (81,4)	ns. (0,062)
**Participation before COVID-19**				
Yes	72 (26,9)	21 (29,2)	51 (70,8)	
No	196 (73,1)	46 (23,5)	150 (76,5)	ns. (0,340)
**Reason for medical consultation**				
Active therapy	218 (83,2)	60 (27,5)	158 (72,5)	
Follow up care	44 (16,8)	6 (13,6)	38 (86,4)	ns. (0,053)
**Type of cancer**				
Solid	124 (51,7)	32 (25,8)	92 (74,2)	
Hematological	116 (48,3)	30 (25,9)	86 (74,1)	ns. (0,992)

Travel time to the hospital (p = 0,003), education level (p < 0,001), occupation (p < 0,001), missed appointments in the past (p = 0,004) and awareness of the definition of eHealth (p = 0,043) were significantly different between the age groups. Compared to the group of older patients, a higher fraction of the younger patients needed less than 30 mins to reach the university hospital. Patients ≥ 55 years old had on average a lower education level than younger patients. Most of the older patients were not employed anymore and the percentage of missed appointments was lower compared to the group of younger patients. No significant age-related difference or trend was observed to community size, frequency of medical consultation, regular medication intake, date of the interview, type of therapy and type of cancer.

**Table 2:** Multiple logistic regressions. Associations between sociodemographic factors, education, insurance status, medication intake, COVID-19 pandemic, reasons for medical consultation with patients’ attitude towards potential of eHealth for intersectoral care in oncological patients.

		Use of appointment reminders	Use of appointment scheduling	Transfer of personal medical information (receive)	Transfer of personal health information (send)	Physician -patient relation-ship	Treatment quality	No concerns about data security	Overall potential
		OR	OR	OR	OR	OR	OR	OR	OR
		(95% CI)	(95% CI)	(95% CI)	(95% CI)	(95% CI)	(95% CI)	(95% CI)	(95% CI)
		Total: N = 206 +: n = 159	Total: N = 206 +: n = 159	Total: N = 239 +: n = 146	Total: N = 239 +: n = 98	Total: N = 238 +: n = 143	Total: N = 239 +: n = 132	Total: N = 237 +: n = 122	Total: N = 232 +: n = 139
**Gender**	Female Male	1 1,69 (0,84–3,39) *(p = 0*,*140)*	1 1,96 (0,97–3,99) *(p = 0*,*062)*	1 2,90 (1,15–3,95) *(p = 0*,*001)*	1 0,97 (0,56–1,69) (p = 0,917)	1 0,91 (0,50–1,65) (p = 0,907)	1 1,71 (0,97–3,00) (p = 0,063)	1 0,88 (0,52–1,49) (p = 0,640)	1 1.47 (0,82–2,65) (p = 0,195)
**Age**	≤ 54 ≥ 55	1 0,55 (0,23–1,34) (p = 0,190)	1 0,82 (0,35–1,95) (p = 0,659)	1 0,52 (0,24–1,11) (p = 0,092)	1 1,08 (0,55–2,13) (p = 0,815)	1 0,52 (0,24–1,12) (p = 0,522)	1 0,51 (0,25–1,04) (p = 0,063)	1 0,69 (0,36–1,34) (p = 0,271)	1 0,48 (0,22–1,03) (p = 0,060)
**Community size (Inhabitants)**	> = 30.000 > 30.000	*1* *1*,*25 (0*,*63–2*,*50)* *(p = 0*,*522)*	1 0,94 (0,47–1,89) (p = 0,866)	1 1,49 (0,82–2,70) (p = 0,191)	1 1,60 (0,78–2,38) (p = 0,282)	1 2,38 (1,30–4,35) (p = 0,005)	1 1,42 (0,81–2,49) (p = 0,227)	1 1,18 (0,69–2,01) (p = 0,547)	1 2,16 (1,19–3,90) (p = 0,011)
**Educational level**	Low Middle +high	1 1,40 (0,63–3,11) *(p = 0*,*404)*	1 1,41 (0,62–3,16) *(p = 0*,*411)*	1 1,84 (0,93–3,63) *(p = 0*,*081)*	1 2,17 (1,08–4,37) *(p = 0*,*031)*	1 1,32 (0,67–2,62) (p = 0,420)	1 1,53 (0,79–2,96) (p = 0,207)	1 0,90 (0,47–1,69) (p = 0,733)	1 1,28 (0,65–2,53) (p = 0,470)
**Employed**	No Yes	1 2,01 (0,79–5,13) *(p = 0*,*142)*	1 1,52 (0,61–3,79) *(p = 0*,*371)*	1 1,15 (0,53–2,47) *(p = 0*,*727)*	1 1,02 (0,52–2,00) (p = 0,954)	1 2,15 (0,97–4,79) (p = 0,061)	1 1,47 (0,72–2,98) (p = 0,290)	1 1,32 (0,68–2,55) (p = 0,414)	1 1,61 (0,75–3,44) (p = 0,224)
**Frequency of medical consultation in the last year**	≤ 5 times > 5 times	1 1,17 (0,44–3,10) *(p = 0*,*748)*	1 1,87 (0,73–4,81) *(p = 0*,*193)*	1 2,19 (0,96–4,98) *(p = 0*,*063)*	1 2,12 (0,87–5,19) (p = 0,099)	1 2,53 (1,08–5,93) (p = 0,033)	1 2,88 (1,25–6,63) (p = 0,013)	1 1,04 (0,48–2,26) (p = 0,930)	1 2,17 (0,95–4,96) (p = 0,067)
**Insurance status**	Statutory health insurance Private health insurance	1 0,90 (0,42–1,94) (p = 0,790)	1 1,35 (0,61–3,01) (p = 0,460)	1 1,95 (0,97–3,91) (p = 0,060)	1 1,20 (0,65–2,23) (p = 0,556)	1 8,87 (0,44–1,71) (p = 0,682)	1 1,26 (0,67–2,39) (p = 0,470)	1 1,17 (0,64–2,13) (p = 0,610)	1 1,32 (0,67–2,60) (p = 0,428)
**Knowledge of the definition of eHealth**	No Yes	1 1,46 (0,64–3,36) (p = 0,379)	1 1,94 (0,80–4,73) (p = 0,144)	1 2,85 (1,31–6,17) *(p = 0*,*008)*	1 2,31 (1,22–4,34) *(p = 0*,*010)*	1 4,37 (1,92–9,97) (p = 0,000)	1 1,77 (0,90–3,50) (p = 0,100)	1 1,52 (0,81–2,85) (p = 0,195)	1 2,54 (1,21–5,34) (p = 0,014)
**Medication intake**	≤ 5 different medication/day ≥ 6 different medication/day	1 1,44 (0,69–3,01) (p = 0,331)	1 0,98 (0,47–2,02) (p = 0,948)	1 1,08 (0,59–1,99) (p = 0,805)	1 0,92 (0,51–1,65) (p = 0,769)	1 1,25 (0,67–2,32) (p = 0,484)	1 0,87 (0,49–1,56) (p = 0,645)	1 0,71 (0,41–1,24) (p = 0,229)	1 0,72 (0,39–1,31) (p = 0,281)
**Participation before COVID-19**	Yes No	1 0,93 (0,42–2,03) (p = 0,845)	1 0,87 (0,39–1,93) (p = 0,732)	1 1,59 (0,84–3,03) (p = 0,157)	1 1,10 (0,60–2,02) (p = 0,761)	1 1,49 (0,77–2,88) (p = 0,238)	1 1,04 (0,56–1,91) (p = 0,910)	1 0,97 (0,54–1,73) (p = 0,913)	1 1,16 (0,62–2,19) (p = 0,644)
**Reasons for medical consultation**	Active therapy Follow up care	1 2,64 (0,83–8,38) (p = 0,099)	1 3,46 (0,95–12,60) (p = 0,060)	1 2,50 (1,00–6,23) (p = 0,050)	1 1,47 (0,67–3,23) (p = 0,335)	1 2,55 (1,01–6,42) (p = 0,048)	1 1,86 (0,81–4,28) (p = 0,143)	1 1,49 (0,69–3,21) (p = 0,314)	1 1,46 (0,61–3,48) (p = 0,399)

### Statistical analysis

The statistical analysis was performed using the IBM Statistical Package for the Social Sciences (SPSS) Version 26 (IBM Corp., Armonk, NY, USA). First, a descriptive analysis was performed for an overview of the answers to the study specific items. A bivariate analysis was used to analyze the relationships between socio-demographic aspects of the study population, the present use of modern ICT, cancer diagnosis as well as the attitude towards eHealth for further use in cross-sectoral care. Cross tabulation was used and Pearsons Chi-square tests were conducted. Responses to the 5-point Likert scales (e.g., statements about eHealth potentials, eHEALS) were transformed to positive (fully, fairly), negative (rather not, not at all) and neutral to analyze statistically significant trends. The study specific results were presented as numbers, percentages, and two tailed p-values. Study participants who did not respond to specific survey questions were excluded from the analysis of these questions.

Multivariate logistic regressions were used to examine the influence of socio-demographic factors on the attitude towards eHealth. The results are expressed as odds ratios (ORs) with a 95% confidence interval (CI). The responses to the questions about patients’ attitude towards eHealth were dichotomized into positive (fully, fairly) and negative (rather not, not at all) and according to the topics, binary response variables were defined. At least half of the corresponding questions had to be answered positively to be rated as positive. Afterwards, one final response variable regarding the overall potential of eHealth was created, which included in total 19 items. 9/19 questions had to be answered positive to consider the question as positive, taking into account the possibility to tick “I don´t know”.

## Results

### Patient characteristics

A total of 305 cancer patients were asked to participate in the survey-based study, which was multicentered at two German university medical centers. 280 patients replied to the survey and 263 of them returned a completed questionnaire. This represents an overall response rate of 86,2% (263/305) and a completion rate of 93,9% (263/280). Both rates are extremely high for this kind of survey-based study. Consequently, the high rates allow for a robust statistical analysis of the study sample [32–36].

#### Socio-demographic factors

48% of our patients were female and 52% male. 25% of the patients were ≤ 54 years old, and 75% were ≥ 55 years old. The mean age of our sample was 61,82 years with a standard deviation of 13,00 years. The study was initiated before the start of the COVID-19 pandemic and 26,9% of the patients participated before the onset of the pandemic. 83,2% of our patients were undergoing an active therapy and 16,8% were in follow up care. The distribution between solid and hematological malignancies was well balanced, 51,7% had a solid tumor and 48,3% had a hematological malignancy. Further results for community size, distance to the university hospital, journey time to the hospital, education level, occupation, frequency of medical consultation, missed appointments, health insurance, awareness of the definition of eHealth and regular medication intake are shown and broken down by the considered ages groups in Table 1.

#### Medication intake and medication plans

The questionnaire also asked for cancer patients’ medication intake and medication plans. As shown in Table 1, 62,8% of our study population had to take 5 or less different medications a day and 37,2% had to take 6 or more medication a day. Fig 1 depicts patients answers to more specific questions about medication. Altogether, 76,6% of the patients had a medication plan, in 96,1% this plan was on paper and only 3,9% had their medication plan as an app (S1 Table). When asked whether they always have their medication plan with them, only 43% answered this question with yes. Patients were also offered eHealth solutions for medication plans, in particular as a smart version (an online based version) or as an app. The former option resulted in 47% approval (37% negation), while for the latter 44% approval (41% negation) was given. Owning a medication plan showed significance to employment (p = 0,001), insurance status (p = 0,038), medication intake (p<0,001), COVID-19 pandemic (p = 0,003) and reasons for medical consultation (p = 0,015). Compared to employed patients a higher fraction of non-employed patients owned a medication plan. 95,8% of patients taking 6 or more different drugs a day owned a medication plan compared to only 65,1% among the patients taking 5 or less drugs a day. Age and education level showed no significance. The use of a medication plan as an app is higher in the group of working participants (p = 0,046). Similar effects were found for patients with a private health insurance (p = 0,025) (S1 Table).

#### Documentation of vital parameters

Patients were asked how they document vital parameters (e.g., blood pressure, weight and temperature) and occurrence of side effects. Results are shown in S2 Table. On average, patients who document their vital parameters digitally or in apps have a higher education level (p<0,001) and are more likely to have a private insurance (p = 0,012). A higher fraction of patients undergoing active therapy reported to have side effects compared to patients in follow up care (p = 0,021). 49,4% of the patients facing side effects contacted their physicians within 48 hours to report them. Patients with hematological tumors reported side effects more often ≤ 48 hours than patients with solid tumors (p = 0,05). To investigate how eHealth can improve side effect management, patients were asked if it is helpful to contact their physicians automatically via app when side effects occur. 47,5% had a positive attitude towards contact via app, 39,1% a negative attitude. Apps to manage side effects could offer services such as push messages asking specific questions like “Did you have a fever during the last days”. 43,0% of patients valued services like this as helpful. Furthermore, less than half of the patients (45,4%) expected improvement of treatment quality through an automatic transfer of side effects via app (S2 Table).

### Dissemination of internet-enabled devices

Another important question was the dissemination of internet-enabled devices as shown in Fig 2A. Age was a significant factor for owning a mobile phone (p≤0,05, see S3 Table). 95,5% of patients ≤ 54 years owned a mobile phone compared to only 79,4% of patients in the age group ≥ 55. Possession of computers with internet access, tablets or smart watches showed no significant age dependency. Education level had a significant impact on possession of all mentioned internet-enabled devices. Patients with a higher education level owned significantly more often any internet-enabled device. A similar effect can be found for knowledge of the definition of eHealth (S3 Table). Date of participation in the study (i.e., prior or during the pandemic), type of cancer and reason for medical consultation showed no significance.

### Penetration and use of modern ICT in daily life

Summarized statistics regarding use of modern ICT in daily life are presented in Fig 2B and S3 Table. There is a significance between age, education level, employment and the importance of internet in private and work life. Of note, employment is associated with age as shown in Table 1. For younger patients (age < = 54 years), internet is more important for their private and work life as compared to patients ≥ 55 years. Patients having a higher education level answered more often that internet use is important for their private and work life than patients with a lower education level. Daily internet usage and daily checking of e-mails both show significance with age. A higher fraction of younger patients were members in social networks compared to older patients (p≤0,001). Patients with a higher education level were significantly more often members of social networks. The COVID-19 pandemic, type of cancer and reason for medical consultation showed no significance.

Besides questions regarding penetration and use of modern ICT in daily life, patients were also asked if they use the internet for online services in daily life other than health related topics such as online shopping and online banking, which was responded with yes by 87,8%. Fig 2C and S4 Table give a detailed overview of the used online services. A higher fraction of patients with a higher education level and occupational activity uses the internet for online banking (p<0,001; p = 0,029), flight/hotel/travel booking (p<0,001; p = 0,06) or news search (p<0,001; p = 0,012).

Interestingly, the COVID 19 pandemic and type of cancer showed significance on non-health-related internet usage. 60,8% of patients who participated in the study during the pandemic answered that they used the internet to search for news compared to only 44,4% of the patients asked before the pandemic (p = 0,016). A similar result was shown for searching for general topics (p = 0,05) or social media as information source. On average, patients asked during COVID-19 used social media more often as information source than patients asked before COVID-19 (p = 0,041, Fig 2D).

### Online health-related information search

Although 87,8% responded that they use online services for daily life (non-health-related topics), only 50,6% of the patients indicated the use of online services for health-related information search. Patients searching health-related information online are significantly younger (p = 0,010, 65,7% ≤ 54 years) and have a higher education level (p<0,001). The kind of health-related information searched for is shown in Fig 3A and S5 Table. Searching for information about medication, healthy lifestyle and nutrition shows significance with age and knowledge of the definition of eHealth. Notably, a higher percentage of patients who search information for physician- and hospital-ranking, are privately insured or have a private additional insurance (p = 0,049). The COVID-19 pandemic situation showed no significance for online health-related information search. Fig 3B provides information on what kind of sources patients use for their health-related information search.

Analyzing the online information sources (results see S6 Table), a significant interrelationship was found between age and the usage of sources blogs (p < 0,001), discussion forums (p < 0,001), information of medical societies (p < 0,01) and pharmaceutical companies (p = 0,001). A significantly higher fraction of the younger age group used these information sources. Additionally, compared to the group of patients with a lower education level, patients with a higher education level used more often discussion forums, physicians’ websites and websites of medical societies and pharmaceutical companies as sources of health information search. Medication intake, occupation and community size showed no significance.

### Quality of online sources for health-related information

Patients were also asked to assess the quality of health information sources. An overview of the evaluation of used online sources is shown in Fig 4. A more detailed analysis showed that people having a higher school qualification rated blogs (p = 0,005), discussion forums, (p = 0,008) physicians’ websites (p = 0,008) and medical societies (p < 0,001) more positive than patients with lower school qualification. Similar effects could be found for occupational education. The COVID-19 pandemic situation showed no significant influence on the assessment of the quality of online sources for health-related information (S7 Table).

### eHealth Literacy Scale (eHEALS)

Fig 5 shows how people evaluate online health-related information search. To this end, following the literature, we used eight specific questions assessing how comfortable and confident patients are with their health-related information search [28–30]. There is a significance between nearly every of the eight questions with age and education level. On average younger patients as well as higher educated patients had better skills and felt more comfortable in using modern ICT for health reasons. The COVID-19 pandemic situation, type of cancer and medication intake showed no significance with questions regarding eHEALS. Detailed results of all questions can be found in S7 Table.

### Attitude towards eHealth

#### Cancer patients’ attitude towards eHealth

Overall, patients had a positive attitude towards eHealth. 66,4% of the patients answered that they would like to receive automatic text message (SMS) or e-mail reminders prior to their appointments. Patients also appreciated the idea of online appointment scheduling (66%), followed by searching online for information prior to a hospital stay (66%), getting medical test results via e-mail (60,6%) and the possibility to use an online chat (56,7%). 42,4% of the patients answered that online video consultation would be helpful, whereas 36,6% responded that they wouldn´t use online video consultation. Less than 20% would send their personal information to their health insurance. 48,1% answered that internet usage improves patient-physician contact and for 48,3% internet usage improves treatment quality. The results are presented in detail in S8 Table.

Multiple logistic regression analyses (Table 2) revealed that men tend to have a slightly more positive attitude towards the overall potential of eHealth than women, especially in terms of receiving medical information (medication plans, referral letters or medical test results) via e-mail, which showed significance (OR 2,90, 95% CI 1,56–5,34). Significance was also shown for community size. Patients living in larger communities (defined as >30.000 inhabitants) are more likely to use online chats and online video consultation than patients living in smaller communities (OR 2,38, 95% CI 1,3–4,35). Living in bigger communities also had a significantly positive impact on the attitude towards the overall potential of eHealth (OR 2,16, 95% CI 1,19–3,9). Cancer patients with a higher school qualification reported a significantly higher willingness to send personal health information to their physician and health insurance (OR 2,17, 95% CI 1,08–4,37). A frequency of medical consultation of more than 5 times in the last year has a significant positive impact regarding patient-physician relationship (using online communication and online video consultation) (OR 2,53, 95% CI 1,08–5,93) and treatment quality (OR 2,882, 95% CI 1,25–6,63). There is no significant difference in patients’ attitude towards eHealth regarding the time they answered the questionnaire (before/during COVID), but the attitude towards the overall potential of eHealth is slightly higher for patients who answered the questionnaire during the COVID-19 pandemic compared to patients who answered it before the start of the pandemic (OR 1,16, 95% CI 0,62–2,19). Also, the attitude towards patient-physician relationship such as online communication and video consultation (OR 1,49, 95% CI 0,77–2,88) as well as receiving personal medical information (OR 1,59, 95% CI 0,84–3,03) was more positive during COVID-19 pandemic. Furthermore, being in follow up care shows a significant influence to physician-patient relationship in comparison to undergoing active therapy (OR 2,55, 95% CI 1,01–6,42).

#### Data security

Concerns about data security without exchange of medical information (e.g., online appointment scheduling) was found in 19% of the patient’s answers (S9 Table). 68,3% have no concerns about data security (without exchange of medical information) and 12,7% don´t know whether they have concerns. The highest concerns about data security in patient-physician interaction (with exchange of medical information) was found with regard to the use of messenger services (47,2%). Less concerns were found for using health apps (36,3%), e-mail (31,7%) or online video consultation (30,3%). On average, the group of younger patients reported to have more concerns about data security than older patients. A higher fraction of study participants living in large communities had data security concerns than participants living in smaller communities.

## Discussion

This study provides an overview of the potential of eHealth from the perspective of outpatients in the special field of haematology-oncology. Collection of data started in September 2019, when COVID-19 was not known yet and lasted one year during the COVID-19 pandemic. This provided the opportunity to explore the potential impact of the corona pandemic on the patients’ attitude towards eHealth. Regardless of the corona pandemic, internet use has been growing rapidly during the last decade and gained increasing importance in private and business life [1]. The COVID-19 pandemic confronted patients, physicians and hospitals with challenges that have never occurred before. eHealth and teleoncology were already known before the pandemic, but suddenly new structures needed to be implemented rapidly [16–18].

During the past 20 years, several studies showed that modern ICT are attracting rising significance for health reasons and change increasingly the health care systems and patient-physician interaction [2, 8, 9]. A study from 2020 revealed that new strategies originally implemented due to COVID-19 pandemic, especially online communication, can be expected to have a high impact beyond everyday life. Most patients wished to continue to use telemedicine services [16–18].

Despite the exceptional relevance of eHealth, especially in the time of the pandemic, little is known about the dissemination of internet-enabled devices, penetration and use of modern ICT in daily life, search for health-related information, attitude towards eHealth and data security concerns of hematological-oncological patients. To close this knowledge gap, 305 patients were asked to answer a specific questionnaire and 280 patients replied to the survey. The total median age in our population was higher than the median age in Germany (61,8 compared to 47,8 years) [37]. The higher median age of the study population is related to a higher median age of cancer patients. The median age in 2018 for a cancer diagnosis was 69 years for women and 70 years for men (Germany) [22]. Dissemination of internet-enabled devices within the study population was high. Most patients (82,4%) reported to have a computer with internet access. Nearly the same percentage (81,9%) owns a smartphone, which is similar to the reported numbers of recently done studies in different patient populations [21, 24]. Slightly more than half of the study population (55,4%) reported to own a tablet and only 10,9% a smartwatch. Regarding the socio-demographic factors it is noteworthy that school qualification as well as age showed significance for owning all of the mentioned internet-enabled devices with higher rates in younger and well-educated patients. These observations go in line with existing studies outside the field of hematology-oncology [21, 24].

Additionally, penetration of modern ICT in everyday life and at work was examined. For 69%, the internet is important for daily life while only 47,5% answered that internet is important for work. That may partially be explained by the median age of 62 years. By taking a closer look at the group of younger patients, the results show that 82,1% rate the internet as important for daily life and 70,1% rate it as important for work life. 70,2% use the internet daily, which appears low compared to the internet penetration of 96% in Germany. The higher average age of the study population is likely to attribute to this result. One conclusion we draw from these results is that the examined patient population might have fewer routines in internet use and therefore potentially needs more support for the use of eHealth applications. 34,7% of the respondents were members of a social network, which had a significant dependency with age. A higher fraction of patients younger than 55 years were members in social networks. This important aspect needs to be addressed for the potential expansion of online support groups in the field of hematology-oncology. We assume that there is great potential especially for younger cancer patients, which should be addressed in future studies.

60,8% of patients interviewed during COVID-19 answered that they search for news on the internet, whereas only 44,4% searched for news online before the pandemic. Even if these results were not significant, the corona pandemic seemed to push digitalization also in hematological-oncological patients.

Only half of the study population (50,6%) used modern ICT for online health-related information search. In contrast, 87,8% used the internet for topics of daily life. The current study showed significance for age and school qualification regarding health-related information search which is similar to previously done studies [38]. Although in the present study not investigated, the accessibility, the trustworthiness and the usability of online available health-related information for hematological-oncological patients must be discussed as potential factors of influence for the study results and need to be further investigated in future studies. Physicians’ websites and homepages of medical societies were the main information sources. This supports the hypothesis that health-related information presented there is considered trustworthy and healthcare professionals should especially support high quality as well as easy accessibility and comprehensibility of health-related information for hematological-oncological patients. Results similar to our study were found in a Dutch study, where 88% reported to use websites of their oncologist, 70% websites of their hospital and 76% the website of the Dutch Cancer Society [38]. A higher fraction of patients with a higher school qualification used these information sources in comparison to patients with lower school qualification. Potential reasons for this might be that information on physicians’ websites and homepages of medical societies might be too complex or that it is hard to find where to get reliable health-related information online. Slightly more of the lower educated patients use social media (13,9%) as health-related information source than higher educated patients (11,1%). Added interesting results were found on questions about eHEALS [28, 29]: 67,8% of the study population said that they have the skills to evaluate the information they find on the internet, but only 27,7% feel confident in using information from the internet to make health decisions. In a former study similar results were found for patients in solid organ transplant care [21]. In a study from 2006, only 24% had the opinion that the use of internet for online health-related information search improves the patient-physician relationship. This value is doubled in our study, which shows that in recent years the patients’ acceptance to online health-related information search significantly increased [39]. Additional future studies are needed, to address the physicians’ attitude towards online available health-related information and their view on how patients’ process that information.

Regarding the attitude towards eHealth, 66,4% of the hematological-oncological patients would like to get text message or e-mail reminders prior to their appointments. Two thirds of the respondents have a positive attitude towards online appointment scheduling as well as online information search prior to a hospital stay. However, eHealth offers far more potential beyond scheduling such as transfer of medical information, online medication plans, online chats and video consultations with physicians and their staff etc.. There is also a high willingness (60,6%) for getting medical test results via e-mail. This shows that the patients’ attitude towards eHealth for cross-sectoral appointment scheduling and transfer of personal medical information is good. Furthermore, these results show the high potential for process improvement by digitalization on the site of the healthcare providers. Further studies with cancer patients confirmed that they feel ready to use eHealth applications such as receiving test results or getting access to their own medical file [38, 40]. Slightly more than half of the patients would use online chats with their physicians and 42,3% would use video calls. A possible reason for the low video call acceptance might be the fear of a lack of personal interaction in video calls. Data security appears to be only of minor relevance as only 30,4% responded that they have concerns about data security when having video calls. A study conducted in Pennsylvania showed that once patients tried medical video calls, satisfaction was high and they were willing to continue using video calls for medical aspects [41]. The radiation oncology department of the University of Texas already used online video consultation before COVID-19 pandemic, mainly to check after Texas Department of Criminal Justice inmates for cancer therapy side effects and follow up care and was quickly able to offer this telemedicine service to other patients in the pandemic [42]. Nearly half of our patient cohort thinks that internet usage improves patient-physician interaction and improves treatment quality. While the current study focuses on the patients’ view on the potential of eHealth, future studies should assess, which types of appointments are suitable for a telemedicine model, in which situations telemedicine actually meet their needs and which conditions necessitate a face-to-face appointment consultation. Concerns due to data security are high when medical information is involved, which is similar to former studies [21, 24]. Concerns about data security without transfer of medical information, however, are low (19%). This underlines that especially administrative processes without the transfer of sensitive medical information can easily be digitized. The questionnaire does not allow further conclusions as to why younger patients are more concerned about data security than older ones. One hypothesis for this finding is that younger patients have more online experience and are better informed about the extent of data misuse and its potential damage than older patients. 47% of hematological-oncological patients are willing to use their medication plan as a smart version and 41% would use a medication plan app. Medication plan apps might not only document medication intake schemes, but could also integrate reminder functions for medication intake. Similar results as for medication plans could be found for documentation and transmission of side effects. More than 40% would like to have an app for contacting their physicians while having side effects and nearly the same number of hematological-oncological patients think that automatic transmission of side effects improves treatment quality. It is conceivable that in addition to an app also chat functions with physicians could improve side effect management and by this improve quality of care.

Besides analyzing dissemination of internet-enabled devices, penetration and use of modern ICT in daily life, online health-related information search and attitude towards eHealth, the study’s aim was to analyze whether the COVID-19 pandemic changed the attitude towards use of telemedicine services. Overall, the date of the interview showed nearly no significant influence in any of the questions. However, even if not significant at conventional levels, the attitude towards the overall potential of eHealth is higher on trend for patients who answered the questionnaire during COVID-19 pandemic (OR 1,46, 95% CI 0,61–3,48). Moreover, the attitude towards the use of online communication and online video consultation tends to be slightly more positive during COVID-19 pandemic (OR 1,487, 95% CI 0,77–2,88). Similar results regarding online video consultation were found in a study at Ohio State University [19].

There are some limitations in this study, which must be considered when interpreting the results. The questionnaire for the interview had 12 pages and it took 15–20 minutes to finish. Most of the hematological-oncological patients, especially when undergoing active therapy, are seriously ill and therefore it was sometimes hard to answer all the questions for some patients. Additionally, nearly three quarters of the study population were interviewed during the COVID-19 pandemic, but the questionnaire was designed before the pandemic. Thus, specific questions regarding COVID-19 and its effect on eHealth were missing. In follow-up studies it is very important to include questions considering the COVID-19 pandemic such as “Did your use of modern ICT (in private or work life) increase during the pandemic?”, “Did your attitude towards telemedicine change during COVID-19 pandemic?” and “Have you increased your search for telemedicine offers during the COVID-19 pandemic?”. However, our study generated an excellent database for further digitalization of cross sectoral care in in the field of haematology-oncology.

In conclusion, this study demonstrates that penetration and use of modern ICT in daily life of oncological patients is high and the attitude and willingness to use eHealth applications for cross-sectoral care exists. Especially automated appointment reminders, online appointment scheduling and getting medical test results via e-mail show a high acceptance rate among our study population. However, the study also shows that only 50,6% of hematological-oncological patients use modern ICT for health-related online search and concerns about data security are still high when exchange of medical information is included. Future research should also focus on data security concerns. It needs to be investigated why patients still are so skeptical when medical information is exchanged, while concerns for e.g. online appointment scheduling is already low. In addition to the investigation of patients`concerns regarding data security, the trustworthiness and usability of online health-related information should be part of future research. Another important aspect is to address the perspective of physicians and nurses with regards to data security concerns and to improve their knowledge and abilities in using eHealth. There might be a need for new professions in medicine to enable nurses, doctors and therapists to better use eHealth. For example, the profession and field of nursing informatics, which is the interface of medical information and technology and exists in the USA and Canada, does not exist in Germany. It is not only essential for nurses and physicians to learn how to use eHealth applications, but also elderly and lower educated patients should get the possibility to learn how to use eHealth applications correctly. Returning to our opening statement “Does COVID-19 pandemic change the attitude towards use of telemedicine services” our analyses show that date of the interview regarding COVID-19 (before or during) revealed almost no significant influence. This leads to the assumption that hematological-oncological patients already have had a positive attitude towards eHealth even before the beginning of the pandemic. Nevertheless, the pandemic required a fast implementation of new eHealth structures for oncological patients. In the future, these structures should be further extended to improve cross-sectoral care and patient-physician relationships in the upcoming years and further studies should focus on the development of new digital treatment pathways for cross sectoral care in hematology-oncology.

## Supporting information

S1 Table Medication plan and medication intake. (PDF)

S2 Table Documentation of vital parameters and side effects. (PDF)

S3 Table Dissemination of internet-enabled devices and penetration. (PDF)

S4 Table Use of modern ICT in daily life. (PDF)

S5 Table Online health related information search. (PDF)

S6 Table Sources of online health information. (PDF)

S7 Table Assessment of online searches for health information by patients eHEALS. (PDF)

S8 Table Cancer patients´ attitude towards eHealth. (PDF)

S9 Table Data security concerns. (PDF)
