# Supplementary material for: The potential of eHealth for cancer patients–does COVID-19 pandemic change the attitude towards use of telemedicine services?
Source: PLoS One. 2023 Feb 10;18(2):e0280723. doi: 10.1371/journal.pone.0280723 (PMC9917238; doi:10.1371/journal.pone.0280723)
Supplement: S1 Table — (PDF) [file pone.0280723.s001.pdf]

		% Usage				
		**Own medication plan**	**Form of use of medication plan: paper vs. digital**	**Always have medication plan with them**	**Medication plan as a „smart version" makes sense**	**Medication plan via app desired**
		Total:<br>N = 265<br>Yes: n = 203	Total:<br>N = 204<br>Digital: n = 8<br>Paper: n = 196<br>(Values in column for paper)	Total:<br>N = 260<br>Yes: n = 112<br>No: n = 105<br>**Don´t have one: n = 43**	Total:<br>N = 264<br>Yes: n = 125<br>No: n = 98<br>I.d.k..: n = 41	Total:<br>N = 264<br>Yes: n = 109<br>No: n = 117<br>I.d.k..: n = 38
**Gender**	Female<br>Male	94 (75,2)<br>106 (77,4)<br>*(p = 0,679)*	90 (96,8)<br>104 (95,4)<br>*(p = 0,621)*	49 (39,5)<br>62 (46,3)<br>*(p = 0,549)*	55 (44,4)<br>70 (50,7)<br>*(p = 0,536)*	49 (39,5)<br>60 (43,5)<br>*(p = 0,401)*
**Age**	≤ 54<br>≥ 55	46 (69,7)<br>151 (78,2)<br>*(p = 0,160)*	42 (91,3)<br>150 (97,4))<br>*(p = 0,064)*	20 (32,3)<br>89 (46,1)<br>*(p = 0,157)*	40 (60,6)<br>84 (43,3)<br>***(p = 0,044)***	36 (54,5)<br>72 (37,1)<br>***(p = 0,044)***
**Community size (Inhabitants)**	>= 30.000<br>> 30.000	100 (75,2)<br>96 (78,7)<br>*(p = 0,508)*	99 (98,0)<br>90 (93,8)<br>*(p = 0,129)*	56 (42,4)<br>53 (44,5)<br>*(p = 0,706)*	58 (43,3)<br>63 (52,5)<br>***(p = 0,068)***	44 (32,8)<br>60 (50,0)<br>***(p = 0,020)***
**Proximity to university hospital**	≤ 20 km<br>≥ 21 km	102 (79,1)<br>98 (74,2)<br>(p = 0,357)	95 (94,1)<br>98 (98,0)<br>(p = 0,153)	52 (40,6)<br>59 (45,7)<br>(p = 0,411)	58 (45,0)<br>67 (51,1)<br>(p = 0,100)	49 (38,0)<br>60 (45,8)<br>(p = 0,272)
**Travel time to hospital**	≤ 30 min<br>≥ 31 min	107 (78,1)<br>91 (74,6)<br>(p = 0,506)	98 (94,2)<br>94 (97,9)<br>(p = 0,184)	54 (39,7)<br>56 (47,1)<br>(p = 0,478)	62 (44,9)<br>63 (52,1)<br>(p = 0,501)	61 (44,2)<br>47 (38,8)<br>(p = 0,623)
**Educational level**	Low<br>Middle + high	62 (78,5)<br>134 (75,3)<br>*(p = 0,578)*	63 (100)<br>127 (94,8)<br>*(p = 0,065)*	30 (39,0)<br>78 (44,3)<br>*(p = 0,594)*	28 (36,4)<br>97 (54,2)<br>*(p=0,030)*	24 (31,2)<br>83 (46,4)<br>*(p=0,039)*
**Occupational level**	Low<br>Middle + high	15 (62,5)<br>181 (77,7)<br>*(p = 0,096)*	16 (100)<br>174 (96,1)<br>***(p = 0,423)***	10 (41,7)<br>99 (43,2)<br>*(p = 0,187)*	5 (21,7)<br>120 (51,3)<br>*(p=0,024)*	7 (30,4)<br>100 (42,7)<br>*(p = 0,521)*
**Employed**	No<br>Yes	158 (81,4)<br>40 (61,5)<br>***(p = 0,001)***	152 (97,4)<br>39 (90,7)<br>***(p = 0,046)***	87 (45,3)<br>23 (36,5)<br>*(p = 0,280)*	86 (44,6)<br>39 (60,0)<br>***(p = 0,038)***	71 (36,8)<br>36 (55,4)<br>***(p = 0,014)***
**Full time or part time job**	≤ 50%<br>> 50 %	18 (78,3)<br>24 (54,5)<br>*(p = 0,057)*	18 (100)<br>23 (85,2)<br>***(p = 0,087)***	6 (26,1)<br>16 (38,1)<br>*(p = 0,140)*	14 (60,9)<br>26 (59,1)<br>*(p = 0,541)*	12 (52,2)<br>24 (54,5)<br>*(p = 0,102)*
**Frequency of medical consultation in the last year**	≤ 5 times<br>> 5 times	31 (72,1)<br>167 (77,3)<br>*(p = 0,461)*	28 (93,3)<br>164 (97,0)<br>*(p = 0,310)*	17 (41,5)<br>91 (42,7)<br>*(p = 0,575)*	12 (29,3)<br>111 (51,2)<br>***(p = 0,008)***	12 (29,3)<br>95 (43,8)<br>*(p = 0,139)*
**Missed appointments in the past**	No<br>Yes	184 (77,3)<br>17 (68,0)<br>*(p = 0,297)*	176 (96,7)<br>17 (89,5)<br>*(p = 0,125)*	103 (44,4)<br>8 (32,0)<br>*(p = 0,492)*	109 (46,6)<br>14 (53,8)<br>*(p = 0,254)*	95 (40,6)<br>13 (50,0)<br>*(p = 0,310)*
**Insurance status**	Statutory health insurance<br>Private health insurance	143 (80,3)<br>57 (68,7)<br>***(p = 0,038)***	143 (97,0)<br>51 (91,1)<br>***(p = 0,025)***	81 (45,5)<br>30 (37,5)<br>*(p = 0,420)*	82 (45,8)<br>42 (51,2)<br>*(p = 0,571)*	69 (38,5)<br>39 (47,6)<br>*(p = 0,079)*
**Knowledge of the definition of eHealth**	Nein<br>Ja	151 (76,3)<br>50 (76,9)<br>*(p = 0,913)*	147 (97,4)<br>47 (92,2)<br>*(p = 0,100)*	80 (41,5)<br>31 (47,7)<br>*(p = 0,679)*	82 (41,8)<br>43 (65,2)<br>***(p = 0,004)***	71 (36,2)<br>38 (57,6)<br>*(p=0,010)*
**Medication intake**	≤ 5 different medication/day<br>≥ 6 different medication/day	108 (65,1)<br>92 (95,8)<br>***(p < 0,001)***	104 (97,2)<br>88 (94,6)<br>***(p = 0,354)***	48 (30,0)<br>62 (63,9)<br>***(p < 0,001)***	75 (46,0)<br>49 (50,5)<br>*(p = 0,416)*	68 (41,7)<br>40 (41,2)<br>*(p = 0,992)*
**Participation before COVID-19**	Yes<br>No	65 (89,0)<br>138 (71,9)<br>(p = 0,003)	64 (98,5)<br>132 (95,0)<br>(p = 0,230)	36 (50,0)<br>76 (40,4)<br>(p = 0,033)	32 (43,8)<br>93 (48,7)<br>(p = 0,778)	25 (34,2)<br>84 (44,0)<br>(p = 0,355)
**Reasons for medical consultation**	Active therapy<br>Follow up care	173 (79,4)<br>26 (61,9)<br>(p = 0,015)	169 (96,6)<br>24 (92,3)<br>(p = 0,299)	99 (45,8)<br>12 (30,8)<br>(p = 0,174)	97 (44,7)<br>26 (61,9)<br>(p = 0,104)	83 (38,2)<br>24 (57,1)<br>(p = 0,030)
**Type of cancer**	Solid<br>Hematological	97 (77,0)<br>90 (78,3)<br>(p = 0,812)	4 (4,1)<br>3 (3,4)<br>(p = 0,798)	52 (41,3)<br>52 (46,4)<br>(p = 0,673)	60 (47,2)<br>58 (50,9)<br>(p = 0,655)	49 (38,6)<br>55 (48,2)<br>(p = 0,316)

S1 Table. Medication plan and medication intake.
