# Supplementary material for: The potential of eHealth for cancer patients–does COVID-19 pandemic change the attitude towards use of telemedicine services?
Source: PLoS One. 2023 Feb 10;18(2):e0280723. doi: 10.1371/journal.pone.0280723 (PMC9917238; doi:10.1371/journal.pone.0280723)
Supplement: S2 Table — (PDF) [file pone.0280723.s002.pdf]

		Documentation of vital parameters and side effects				
		**App contact with physician in case of side effects.**	**Automatic transmissions of side effects via app. Treatment quality.**	**How to document vital parameters (paper. vs. digital vs. app vs. not)**	**Side effects during therapy.**	**Waiting time for contacting physician <=48h**
		Total:<br>N = 263<br>pos: n = 125<br>neg: n = 103<br>I.d.k..: n = 35	Total:<br>N = 264<br>pos: n = 120<br>neg: n = 100<br>I.d.k.: n = 44	Total:<br>N = 231<br>paper: n = 125<br>digital: 15<br>app: 9<br>not: n = 82<br>(Values in column for paper)	Total:<br>N = 266<br>Yes: n = 144	Total:<br>N = 180<br>Yes: n = 89
**Gender**	Female<br>Male	58 (46,8)<br>67 (48,6)<br>(p = 0,958)	54 (43,2)<br>66 (47,8)<br>(p = 0,752)	59 (55,1)<br>65 (52,8)<br>(p = 0,303)	67 (52,8)<br>76 (55,1)<br>(p = 0,705)	40 (47,6)<br>50 (52,6)<br>(p = 0,503)
**Age**	≤ 54<br>≥ 55	41 (61,2)<br>83 (43,0)<br>(p = 0,028)	41 (61,2)<br>78 (40,2)<br>(p = 0,012)	22 (40,0)<br>101 (58,4)<br>(p = 0,057)	52 (78,8)<br>90 (45,7)<br>(p < 0,001)	32 (59,3)<br>56 (45,2)<br>(p = 0,084)
**Community size (Inhabitants)**	>= 30.000<br>> 30.000	57 (42,5)<br>61 (50,4)<br>(p = 0,395)	56 (41,5)<br>58 (47,9)<br>(p = 0,584)	72 (61,5)<br>48 (44,4)<br>(p = 0,052)	71 (52,2)<br>68 (55,7)<br>(p = 0,570)	46 (50,5)<br>40 (48,2)<br>(p = 0,756)
**Proximity to university hospital**	≤ 20 km<br>≥ 21 km	63 (48,1)<br>61 (46,9)<br>(p = 0,850)	60 (45,8)<br>60 (45,8)<br>(p = 0,442)	60 (53,6)<br>64 (54,2)<br>(p = 0,469)	72 (55,0)<br>71 (53,8)<br>(p = 0,848)	38 (42,2)<br>51 (57,3)<br>(p = 0,044)
**Travel time to hospital**	≤ 30 min<br>≥ 31 min	69 (49,6)<br>54 (44,6)<br>(p = 0,549)	64 (45,7)<br>55 (45,5)<br>(p = 0,339)	58 (49,2)<br>65 (59,1)<br>(p = 0,508)	82 (59,0)<br>61 (49,6)<br>(p = 0,127)	51 (49,0)<br>37 (50,0)<br>(p = 0,899)
**Educational level**	Low<br>Middle + high	26 (33,8)<br>96 (53,3)<br>(p = 0,010)	25 (32,1)<br>93 (51,7)<br>(p = 0,006)	53 (74,6)<br>71 (45,2)<br>(p<0,001)	37 (46,3)<br>105 (58,3)<br>(p = 0,071)	25 (48,1)<br>64 (51,2)<br>(p = 0,705)
**Occupational level**	Low<br>Middle + high	11 (45,8)<br>112 (47,9)<br>(p = 0,952)	11 (44,0)<br>108 (46,2)<br>(p = 0,707)	14 (66,7)<br>110 (53,1)<br>(p = 0,649)	10 (41,7)<br>132 (55,9)<br>(p = 0,181)	9 (60,0)<br>80 (49,4)<br>(p = 0,431)
**Employed**	No<br>Yes	85 (44,0)<br>38 (57,6)<br>(p = 0,154)	80 (41,2)<br>39 (59,1)<br>(p = 0,012)	102 (58,6)<br>22 (40,0)<br>(p = 0,053)	104 (52,8)<br>39 (60,0)<br>(p = 0,311)	65 (48,9)<br>24 (53,3)<br>(p = 0,605)
**Full time or part time job**	≤ 50%<br>> 50 %	12 (50,0)<br>26 (59,1)<br>(p = 0,771)	12 (50,0)<br>28 (63,6)<br>(p = 0,436)	10 (50,0)<br>14 (36,8)<br>(p = 0,246)	15 (62,5)<br>24 (55,8)<br>(p = 0,595)	9 (56,3)<br>15 (51,7)<br>(p = 0,771)
**Frequency of medical consultation in the last year**	≤ 5 times<br>> 5 times	16 (39,0)<br>106 (48,4)<br>(p = 0,366)	14 (34,1)<br>103 (47,0)<br>(p = 0,128)	26 (76,5)<br>95 (49,5)<br>(p = 0,031)	15 (35,7)<br>128 (58,4)<br>(p = 0,007)	11 (40,7)<br>77 (51,3)<br>(p = 0,311)
**Missed appointments in the past**	No<br>Yes	111 (47,6)<br>12 (44,4)<br>(p = 0,946)	107 (45,7)<br>11 (40,7)<br>(p = 0,413)	111 (52,6)<br>12 (66,7)<br>(p = 0,339)	126 (53,6)<br>15 (55,6)<br>(p = 0,848)	76 (48,7)<br>13 (61,9)<br>(p = 0,257)
**Insurance status**	Statutory health insurance<br>Private health insurance	79 (44,1)<br>45 (54,9)<br>(p = 0,153)	76 (42,5)<br>43 (51,8)<br>(p = 0,296)	92 (58,2)<br>31 (43,7)<br>(p = 0,012)	97 (53,0)<br>45 (55,6)<br>(p = 0,702)	57 (47,9)<br>32 (54,2)<br>(p = 0,426)
**Knowledge of the definition of eHealth**	Nein<br>Ja	86 (43,9)<br>39 (58,2)<br>(p = 0,023)	83 (42,1)<br>37 (55,2)<br>(p = 0,158)	103 (59,9)<br>22 (37,3)<br>(p = 0,012)	102 (52,0)<br>42 (62,7)<br>(p = 0,131)	67 (49,6)<br>22 (48,9)<br>(p = 0,931)
**Medication intake**	≤ 5 different medication/day<br>≥ 6 different medication/day	82 (50,6)<br>42 (42,9)<br>(p = 0,389)	77 (47,2)<br>42 (42,9)<br>(p = 0,587)	68 (47,6)<br>56 (65,1)<br>(p = 0,076)	86 (52,1)<br>57 (58,8)<br>(p = 0,297)	54 (49,1)<br>34 (50,0)<br>(p = 0,906)
**Participation before COVID-19**	Yes<br>No	30 (41,1)<br>95 (50,0)<br>(p = 0,285)	30 (41,1)<br>90 (47,1)<br>(p = 0,639)	35 (53,8)<br>90 (54,2)<br>(p = 0,675)	46 (63,0)<br>98 (50,8)<br>(p = 0,074)	29 (56,9)<br>60 (46,5)<br>(p = 0,211)
**Reasons for medical consultation**	Active therapy<br>Follow up care	99 (45,4)<br>25 (61,0)<br>(p = 0,169)	95 (43,4)<br>24 (58,5)<br>(p = 0,082)	105 (54,7)<br>19 (51,4)<br>(p = 0,925)	126 (57,5)<br>16 (38,1)<br>(p = 0,021)	75 (50,3)<br>13 (48,1)<br>(p = 0,834)
**Type of cancer**	Solid<br>Hematological	53 (42,1)<br>61 (53,5)<br>(p = 0,195)	52 (40,9)<br>57 (50,0)<br>(p = 0,220)	63 (56,8)<br>53 (53,0)<br>(p = 0,439)	73 (57,9)<br>60 (52,6)<br>(p = 0,409)	38 (42,7)<br>43 (58,1)<br>(p = 0,050)

S2 Table. Documentation of vital parameters and side effects.
