# Supplementary material for: The potential of eHealth for cancer patients–does COVID-19 pandemic change the attitude towards use of telemedicine services?
Source: PLoS One. 2023 Feb 10;18(2):e0280723. doi: 10.1371/journal.pone.0280723 (PMC9917238; doi:10.1371/journal.pone.0280723)
Supplement: S3 Table — (PDF) [file pone.0280723.s003.pdf]

		Total n and % of patients using digital information and communication systems.								
		**Importance of internet (work life)**	**Importance of internet (private life)**	**Computer with internet access**	**Possesion of a mobile phone**	**Possesion of a tablet/ iPad**	**Possesion of a smartwatch**	**Member of a social network.**	**Daily internet usage.**	**Daily e-mail usage.**
		Total:<br>N = 265<br>Positive: n = 126	Total:<br>N = 274<br>Positive: n = 189	Total:<br>N = 279<br>Yes: n = 230	Total:<br>N = 277<br>Yes: n = 227	Total:<br>N = 278<br>Yes: n = 154	Total:<br>N = 276<br>Yes: n = 30	Total:<br>N = 277<br>Yes: n = 96	Total:<br>N = 275<br>Yes: n = 193	Total:<br>N = 275<br>Yes: n = 166
**Gender**	Female<br>Male	50 (40,7)<br>74 (54,0)<br>**(p = 0,031)**	82 (65,1)<br>105 (74,5)<br>(p = 0,095)	104 (80,6)<br>125 (88,7)<br>(p = 0,066)	109 (85,8)<br>114 80,9)<br>(p = 0,277)	69 (53,5)<br>83 (59,3)<br>(p = 0,338)	12 (9,5)<br>18 (12,8)<br>(p = 0,402)	43 (33,9)<br>52 (36,9)<br>(p = 0,606)	88 (69,3)<br>104 (74,3)<br>(p = 0,365)	70 (55,1)<br>95 (67,9)<br>(p = 0,032)
**Age**	≤ 54<br>≥ 55	47 (70,1)<br>76 (40,0)<br>**(p < 0,001)**	55 (82,1)<br>131 (66,5)<br>**(p = 0,016)**	62 (92,5)<br>166 (83,0)<br>(p = 0,056)	63 (95,5)<br>158 (79,4)<br>**(p = 0,002)**	44 (65,7)<br>106 (53,3)<br>(p = 0,077)	11 (16,7)<br>19 (9,6)<br>(p = 0,117)	43 (65,2)<br>51 (25,6)<br>**(p < 0,001)**	63 (94,0)<br>128 (65,0)<br>**(p < 0,001)**	52 (77,6)<br>111 (56,3)<br>**(p = 0,002)**
**Community size (Inhabitants)**	>= 30.000<br>> 30.000	57 (43,2)<br>63 (52,5)<br>(p = 0,139)	86(62,8)<br>95 (77,9)<br>**(p = 0,008)**	116 (84,7)<br>108 (87,1)<br>(p = 0,575)	107 (78,7)<br>109 (88,6)<br>(p = 0,032)	66 (48,2)<br>82 (66,7)<br>**(p = 0,003)**	17 (12,5)<br>13 (10,6)<br>(p = 0,628)	44 (32,1)<br>45 (36,6)<br>(p = 0,448)	94 (68,6)<br>92 (74,8)<br>(p = 0,270)	74 (54.0)<br>86 (69,9)<br>(p = 0,008)
**Proximity to university hospital**	≤ 20 km<br>≥ 21 km	65 (49,6)<br>59 (46,8)<br>(p = 0,654)	94 (71,8)<br>92 (69,2)<br>(p = 0,646)	113 (85,0)<br>115 (85,8)<br>(p = 0,843)	110 (83,3)<br>110 (82,7)<br>(p = 0,892)	7 (55,6)<br>77 (57,9)<br>(p = 0,710)	15 (11,5)<br>15 (11,3)<br>(p = 0,965)	48 (36,4)<br>46 (34,6)<br>(p = 0,762)	93 (69,9)<br>98 (74,2)<br>(p = 0,433)	80 (60,2)<br>84 (63,6)<br>(p = 0,559)
**Travel time to hospital**	≤ 30 min<br>≥ 31 min	73 (54,5)<br>51 (42,1)<br>(p = 0,049)	103 (74,1)<br>82 (66,7)<br>(p = 0,187)	121 (85,8)<br>197 (86,3)<br>(p = 0,911)	117 (83,6)<br>103 (83,1)<br>(p = 0,912)	81 (57,9)<br>69 (55,6)<br>(p = 0,717)	15 (10,9)<br>15 (12,1)<br>(p = 0,755)	51 (36,7)<br>42 (33,9)<br>(p = 0,633)	106 (75,2)<br>84 (68,9)<br>(p = 0,253)	89 (63,1)<br>75 (61,5)<br>(p = 0,784)
**Educational level**	Low<br>Middle + high	24 (30,0)<br>99 (56,3)<br>**(p < 0,001)**	44 (54,3)<br>138 (76,7)<br>**(p < 0,001)**	60 (72,3)<br>165 (91,2)<br>**(p < 0,001)**	55 (67,1)<br>163 (90,6)<br>**(p < 0,001)**	37 (44,6)<br>111 (61,7)<br>**(p = 0,009)**	2 (2,5)<br>28 (15,6)<br>**(p = 0,002)**	15 (18,3)<br>78 (43,3)<br>**(p < 0,001)**	44 (53,7)<br>143 (79,4)<br>**(p < 0,001)**	34 (41,5)<br>127 (70,6)<br>**(p < 0,001)**
**Occupational level**	Low<br>Middle + high	8 (33,3)<br>**115 (49,6)**<br>**(p = 0,130)**	12 (48,0)<br>**171 (72,2)**<br>**(p < 0,012)**	19 (73,1)<br>**207 (87,3)**<br>**(p = 0,047)**	20 (80,0)<br>198 (83,9)<br>(p = 0,617)	9 (36,0)<br>140 (59,1)<br>**(p = 0,027)**	2 (8,0)<br>28 (11,9)<br>(p = 0,565)	6 (24,0)<br>87 (36,7)<br>**(p = 0,207)**	13 (50,0)<br>175 (73,8)<br>**(p = 0,011)**	11 (42,3)<br>**151 (63,7)**<br>**(p = 0,033)**
**Employed**	No<br>Yes	71 (37,4)<br>53 (79,1)<br>**(p < 0,001)**	127 (64,5)<br>57 (86,4)<br>**(p = 0,001)**	163 (81,9)<br>64 (97,0)<br>**(p = 0,002)**	159 (80,7)<br>60 (90,9)<br>(p = 0,055)	105 (53,3)<br>45 (67,2)<br>**(p = 0,048)**	16 (8,1)<br>14 (21,2)<br>**(p = 0,004)**	59 (29,9)<br>34 (50,7)<br>**(p = 0,002)**	129 (65,5)<br>60 (89,6)<br>**(p < 0,001)**	109 (55,3)<br>54 (80,6)<br>**(p < 0,001)**
**Full time or part time job**	≤ 50%<br>> 50 %	18 (72,0)<br>35 (79,5)<br>(p = 0,475)	19 (79,2)<br>38 (86,4)<br>(p = 0,441)	22 (91,7)<br>43 (97,7)<br>(p = 0,245)	21 (87,5)<br>40 (90,9)<br>(p = 0,658)	13 (52,0)<br>33 (75,0)<br>(p = 0,051)	4 (16,7)<br>10 (22,7)<br>(p = 0,555)	10 (40,0)<br>23 (52,3)<br>(p = 0,327)	20 (80,0)<br>39 (88,6)<br>(p = 0,327)	18 (72,0)<br>35 (79,5)<br>(p = 0,475)
**Frequency of medical consultation in the last year**	≤ 5 times<br>> 5 times	15 (37,5)<br>108 (50,2)<br>(p = 0,139)	23 (52,3)<br>163 (75,1)<br>**(p = 0,002)**	33 (73,3)<br>192 (87,7)<br>**(p = 0,014)**	33 (73,3)<br>186 (84,9)<br>(p = 0,060)	18 (40,0)<br>133 (60,7)<br>**(p = 0,010)**	3 (6,8)<br>27 (12,4)<br>(p = 0,290)	12 (27,3)<br>83 (37,9)<br>(p = 0,181)	21 (48,8)<br>169 (77,2)<br>**(p < 0,001)**	15 (34,9)<br>148 (67,6)<br>**(p < 0,001)**
**Missed appointments in the past**	No<br>Yes	110 (47,6)<br>13 (48,1)<br>(p = 0,958)	166 (69,5)<br>20 (74,1)<br>(p = 0,620)	203 (83,9)<br>24 (88,9)<br>(p = 0,497)	202 (84,2)<br>19 (73,1)<br>(p = 0,152)	138 (57,5)<br>14 (51,9)<br>(p = 0,574)	29 (12,1)<br>1 (3,7)<br>(p = 0,189)	78 (32,6)<br>15 (55,6)<br>**(p = 0,018)**	169 (70,7)<br>21 (77,8)<br>(p =0,441)	147 (61,5)<br>16 (59,3)<br>(p =0,820)
**Insurance status**	Statutory health insurance<br>Private health insurance	80 (44,0)<br>43 (55,8)<br>(p = 0,080)	119 (64,7)<br>67 (81,7)<br>**(p = 0,005)**	152 (81,7)<br>76 (91,6)<br>**(p = 0,038)**	151 (81,6)<br>71 (86,6)<br>(p = 0,318)	93 (50,0)<br>58 (70,7)<br>**(p = 0,002)**	19 (10,3)<br>11 (13,6)<br>(p = 0,432)	68 (36,6)<br>26 (32,1)<br>(p = 0,483)	127 (69,4)<br>64 (77,1)<br>(p = 0,195)	102 (55,7)<br>62 (74,7)<br>**(p = 0,003)**
**Knowledge of the definition of eHealth**	No<br>Yes	80 (41,0)<br>45 (69,2)<br>**(p < 0,001)**	128 (64,0)<br>60 (89,6)<br>**(p < 0,001)**	166 (82,6)<br>63 (94,0)<br>**(p = 0,021)**	160 (80,0)<br>63 (95,5)<br>**(p = 0,003)**	107 (53,5)<br>46 (68,7)<br>**(p = 0,030)**	18 (9,0)<br>12 (17,9)<br>**(p =0,047)**	57 (28,5)<br>38 (56,7)<br>**(p < 0,001)**	128 (63,4)<br>65 (97,0)<br>**(p < 0,001)**	108 (53,5)<br>58 (86,6)<br>**(p < 0,001)**
**Medication intake**	≤ 5 different medication/day<br>≥ 6 different medication/day	82 (50,9)<br>41 (42,3)<br>(p = 0,177)	120 (72,7)<br>66 (66,0)<br>(p = 0,246)	143 (85,1)<br>83 (83,0)<br>(p = 0,644)	140 (83,8)<br>81 (81,8)<br>(p = 0,672)	97 (58,1)<br>55 (55,0)<br>(p = 0,622)	22 (13,3)<br>8 (8,0)<br>(p = 0,184)	61 (36,7)<br>33 (33,0)<br>(p = 0,536)	120 (72,3)<br>71 (71,0)<br>(p = 0,821)	104 (62,7)<br>60 (60,0)<br>(p = 0,667)
**Participation before COVID-19**	Yes<br>No	31 (41,9)<br>95 (49,7)<br>(p = 0,251)	55 (75,3)<br>134 (67,0)<br>(p = 0,245)	62 (83,8)<br>168 (82,0)<br>(p= 0,722)	58 (78,4)<br>169 (83,3)<br>(p = 0,351)	44 (59,5)<br>110 (54,9)<br>(p = 0,412)	5 (6,8)<br>25 (12,4)<br>(p = 0,184)	24 (32,4)<br>72 (35,5)<br>(p = 0,639)	54 (74,0)<br>139 (68,8)<br>(p = 0,409)	41 (56,2)<br>125 (61,9)<br>(p = 0,392)
**Reasons for medical consultation**	Active therapy<br>Follow up care	99 (46,3)<br>24 (57,1)<br>(p = 0,197)	152 (69,4)<br>32 (74,4)<br>(p = 0,511)	189 (85,5)<br>37 (84,1)<br>(p = 0,807)	183 (83,6)<br>36 (81,8)<br>(p = 0,777)	127 (57,7)<br>26 (59,1)<br>(p = 0,867)	25 (11,4)<br>5 (11,6)<br>(p = 0,968)	78 (35,5)<br>16 (37,2)<br>(p = 0,826)	159 (71,9)<br>31 (73,8)<br>(p = 0,805)	135 (61,1)<br>28 (66,7)<br>(p = 0,495)
**Type of cancer**	Solid<br>Hematological	55 (44,0)<br>60 (54,1)<br>(p = 0,123)	86 (67,2)<br>87 (76,3)<br>(p = 0,116)	108 (84,4)<br>103 (88,8)<br>(p = 0,314)	108 (85,0)<br>95 (81,2)<br>(p = 0,423)	76 (59,8)<br>71 (61,2)<br>(p = 0,828)	13 (10,3)<br>15 (13,0)<br>(p = 0,509)	48 (38,1)<br>42 (36,2)<br>(p = 0,761)	86 (67,2)<br>93 (80,9)<br>(p = 0,016)	76 (59,4)<br>78 (67,8)<br>(p = 0,172)

S3 Table. Dissemination of internet-enabled devices and penetration.
