# Supplementary material for: The potential of eHealth for cancer patients–does COVID-19 pandemic change the attitude towards use of telemedicine services?
Source: PLoS One. 2023 Feb 10;18(2):e0280723. doi: 10.1371/journal.pone.0280723 (PMC9917238; doi:10.1371/journal.pone.0280723)
Supplement: S4 Table — (PDF) [file pone.0280723.s004.pdf]

		% of patients using the internet in daily life							
		Using online services	Shopping	Banking	Bookings	News	Social Media	Search for general topics	Others
		Total:<br>N = 271<br>Ja: n = 238	Total:<br>N = 270<br>Ja: n = 152	Total:<br>N = 270<br>Ja: n = 141	Total:<br>N = 271<br>Ja: n = 108	Total:<br>N = 271<br>Ja: n = 153	Total:<br>N = 271<br>Ja: n = 73	Total:<br>N = 271<br>Ja: n = 169	Total:<br>N = 271<br>Ja: n = 33
**Gender**	Female<br>Male	110 (87,3)<br>127 (90,7)<br>*(p = 0,373)*	66 (52,4)<br>85 (61,2)<br>*(p = 0,150)*	61 (48,4)<br>79 (56,8)<br>*(p = 0,170)*	49 (38,9)<br>59 (42,1)<br>(p = 0,589)	63 (50,0)<br>89 (63,6)<br>***(p = 0,026)***	30 (23,8)<br>43 (30,7)<br>*(p = 0,208)*	78 (61,9)<br>90 (64,3)<br>*(p = 0,688)*	15 (11,9)<br>18 (12,9)<br>*(p = 0,814)*
**Age**	≤ 54<br>≥ 55	65 (97,0)<br>170 (86,7)<br>***(p = 0,019)***	49 (73,1)<br>102 (52,3)<br>***(p = 0,003)***	42 (62,7)<br>96 (49,2)<br>*(p = 0,057)*	38 (56,7)<br>70 (35,7)<br>**(p = 0,003)**	50 (74,6)<br>101 (51,5)<br>**(p = 0,001)**	31 (46,3)<br>42 (21,4)<br>***(p < 0,001)***	48 (71,6)<br>120 (61,2)<br>*(p = 0,125)*	8 (11,9)<br>25 (12,8)<br>*(p = 0,862)*
**Community size (Inhabitants)**	>= 30.000<br>> 30.000	115 (84,6)<br>115 (93,5)<br>*(p = 0,023)*	70 (51,5)<br>77 (63,1)<br>*(p = 0,059)*	61 (44,9)<br>72 (59,0)<br>***(p = 0,023)***	44 (32,4)<br>63 (51,2)<br>**(p = 0,002)**	60 (44,1)<br>87 (70,7)<br>***(p < 0,001)***	27 (19,9)<br>43 (35,0)<br>***(p = 0,006)***	75 (55,1)<br>90 (73,2)<br>**(p = 0,003)**	22 (16,2)<br>11 (8,9)<br>*(p = 0,081)*
**Proximity to university hospital**	≤ 20 km<br>≥ 21 km	120 (90,2)<br>116 (88,5)<br>(p = 0,658)	76 (57,1)<br>74 (56,9)<br>(p = 0,971)	68 (51,1)<br>71 (54,6)<br>(p = 0,571)	57 (42,9)<br>51 (38,9)<br>(p = 0,517)	81 (60,9)<br>70 (53,4)<br>(p = 0,220)	38 (28,6)<br>35 (26,7)<br>(p = 0,736)	88 (66,2)<br>79 (60,3)<br>(p = 0,323)	14 (10,5)<br>19 (14,5)<br>(p = 0,329)
**Travel time to hospital**	≤ 30 min<br>≥ 31 min	131 (92,9)<br>104 (86,0)<br>(p = 0,065)	82 (58,2)<br>68 (56,7)<br>(p = 0,808)	78 (55,3)<br>61 (50,8)<br>(p = 0,469)	60 (42,6)<br>48 (39,7)<br>(p = 0,636)	89 (63,1)<br>61 (50,4)<br>(p = 0,038)	41 (29,1)<br>31 (25,6)<br>(p = 0,532)	92 (65,2)<br>74 (61,2)<br>(p = 0,493)	19 (13,5)<br>13 (10,7)<br>(p = 0,501)
**Educational level**	Low<br>Middle + high	63 (77,8)<br>169 (93,9)<br>***(p < 0,001)***	35 (43,2)<br>113 (63,1)<br>***(p = 0,003)***	28 (34,6)<br>108 (60,3)<br>***(p < 0,001)***	16 (19,8)<br>90 (50,0)<br>***(p < 0,001)***	26 (32,1)<br>122 (67,8)<br>***(p < 0,001)***	7 (8,6)<br>64 (35,6)<br>***(p < 0,001)***	34 (42,0)<br>129 (71,7)<br>***(p < 0,001)***	10 (12,3)<br>21 (11,7)<br>*(p = 0,875)*
**Occupational level**	Low<br>Middle + high	19 (73,1)<br>214 (90,7)<br>***(p = 0,007)***	11 (42,3)<br>138 (58,7)<br>***(p = 0,109)***	11 (42,3)<br>126 (53,6)<br>***(p = 0,273)***	6 (23,1)<br>101 (42,8)<br>***(p = 0,052)***	12 (46,2)<br>137 (58,1)<br>***(p = 0,245)***	5 (19,2)<br>67 (28,4)<br>*(p = 0,321)*	13 (50,0)<br>151 (64,0)<br>***(p = 0,162)***	0 (0,0)<br>31 (13,1)<br>*(p = 0,049)*
**Employed**	No<br>Yes	170 (86,7)<br>64 (95,5)<br>*(p = 0,047)*	104 (53,3)<br>46 (68,7)<br>***(p = 0,029)***	95 (48,7)<br>43 (64,2)<br>***(p = 0,029)***	71 (36,2)<br>37 (55,2)<br>***(p = 0,006)***	103 (52,6)<br>47 (70,1)<br>***(p = 0,012)***	45 (23,0)<br>27 (40,3)<br>***(p = 0,006)***	114 (58,2)<br>51 (76,1)<br>***(p = 0,009)***	21 (10,7)<br>10 (14,9)<br>*(p = 0,356)*
**Full time or part time job**	≤ 50%<br>> 50 %	22 (88,0)<br>42 (95,5)<br>*(p = 0,251)*	11 (44,0)<br>34 (77,3)<br>***(p = 0,005)***	12 (48,0)<br>31 (70,5)<br>*(p = 0,064)*	9 (36,0)<br>28 (63,6)<br>***(p = 0,027)***	16 (64,0)<br>31 (70,5)<br>*(p = 0,580)*	6 (24,0)<br>21 (47,7)<br>*(p = 0,052)*	16 (64,0)<br>35 (79,5)<br>*(p = 0,158)*	4 (16,0)<br>6 (13,6)<br>*(p = 0,789)*
**Frequency of medical consultation in the last year**	≤ 5 times<br>> 5 times	35 (81,4)<br>199 (91,3)<br>*(p = 0,052)*	18 (41,9)<br>130 (59,9)<br>*(p = 0,029)*	20 (46,5)<br>118 (54,4)<br>*(p = 0,345)*	14 (32,6)<br>92 (42,2)<br>*(p = 0,239)*	17 (39,5)<br>134 (61,5)<br>***(p = 0,008)***	11 (25,6)<br>62 (28,4)<br>*(p = 0,703)*	19 (44,2)<br>147 (67,4)<br>***(p = 0,004)***	1 (2,3)<br>32 (14,7)<br>*(p = 0,026)*
**Missed appointments in the past**	No<br>Yes	211 (88,7)<br>24 (88,9)<br>*(p = 0,971)*	134 (56,5)<br>15 (55,6)<br>*(p = 0,922)*	123 (51,9)<br>15 (55,6)<br>*(p = 0,719)*	96 (40,3)<br>11 (40,7)<br>*(p = 0,968)*	133 (55,9)<br>18 (66,7)<br>*(p = 0,283)*	60 (25,2)<br>11 (40,7)<br>*(p = 0,084)*	150 (63,0)<br>16 (59,3)<br>*(p = 0,701)*	30 (12,8)<br>2 (7,4)<br>*(p = 0,432)*
**Insurance status**	Statutory health insurance<br>Private health insurance	158 (86,8)<br>78 (94,0)<br>***(p = 0,083)***	96 (53,0)<br>54 (65,1)<br>*(p = 0,067)*	85 (47,0)<br>54 (65,1)<br>***(p = 0,006)***	58 (31,9)<br>49 (59,0)<br>***(p < 0,001)***	96 (52,7)<br>55 (66,3)<br>***(p = 0,039)***	49 (26,9)<br>23 (27,7)<br>*(p = 0,894)*	103 (56,6)<br>64 (77,1)<br>***(p = 0,001)***	21 (11,5)<br>12 (14,5)<br>*(p = 0,504)*
**Knowledge of the definition of eHealth**	No<br>Yes	171 (85,1)<br>67 (100,0)<br>***(p = 0,001)***	101 (50,5)<br>51 (76,1)<br>***(p < 0,001)***	92 (46,0)<br>49 (73,1)<br>***(p < 0,001)***	71 (35,3)<br>37 (55,2)<br>***(p = 0,004)***	99 (49,3)<br>54 (80,0)<br>***(p < 0,001)***	41 (20,4)<br>32 (47,8)<br>***(p < 0,001)***	110 (54,7)<br>59 (88,1)<br>***(p < 0,001)***	24 (11,9)<br>9 (13,4)<br>*(p = 0,747)*
**Medication intake**	≤ 5 different medication/day<br>≥ 6 different medication/day	150 (90,9)<br>86 (86,0)<br>*(p = 0,215)*	98 (59,8)<br>52 (52,0)<br>*(p = 0,217)*	87 (53,0)<br>53 (53,0)<br>*(p = 0,994)*	75 (45,5)<br>32 (32,0)<br>**(p = 0,030)**	95 (57,6)<br>56 (56,0)<br>*(p = 0,802)*	49 (29,7)<br>24 (24,0)<br>*(p = 0,314)*	105 (63,6)<br>63 (63,0)<br>*(p = 0,917)*	21 (12,7)<br>12 (12,0)<br>*(p = 0,862)*
**Participation before COVID-19**	Yes<br>No	63 (87,5)<br>175 (87,9)<br>(p = 0,922)	41 (56,9)<br>111 (56,1)<br>(p = 0,897)	38 (52,8)<br>103 (52,0)<br>(p = 0,912)	33 (45,8)<br>75 (37,7)<br>(p = 0,226)	32 (44,4)<br>121 (60,8)<br>(p = 0,016)	14 (19,4)<br>59 (29,6)<br>(p = 0,094)	35 (48,6)<br>134 (67,3)<br>(p = 0,005)	11 (15,3)<br>22 (11,1)<br>(p = 0,348)
**Reasons for medical consultation**	Active therapy<br>Follow up care	197 (89,5)<br>37 (88,1)<br>(p = 0,780)	125 (57,1)<br>26 (61,9)<br>(p = 0,562)	116 (53,0)<br>22 (52,4)<br>(p = 0,944)	88 (40,0)<br>17 (40,5)<br>(p = 0,954)	124 (56,4)<br>28 (66,7)<br>(p = 0,215)	58 (26,4)<br>14 (33,3)<br>(p = 0,354)	139 (63,2)<br>28 (66,7)<br>(p = 0,667)	30 (13,6)<br>2 (4,8)<br>(p = 0,108)
**Type of cancer**	Solid<br>Hematological	115 (90,6)<br>105 (91,3)<br>(p = 0,839)	68 (53,5)<br>77 (67,5)<br>(p = 0,027)	64 (50,4)<br>68 (59,6)<br>(p = 0,149)	46 (36,2)<br>56 (48,7)<br>(p = 0,050)	72 /56,7)<br>71 (61,7)<br>(p = 0,425)	33 (26,0)<br>34 (29,6)<br>(p = 0,534)	79 (62,2)<br>79 (68,7)<br>(p = 0,289)	17 (13,4)<br>15 (13,0)<br>(p = 0,937)

S4: Use of modern ICT in daily life.
