# Supplementary material for: The potential of eHealth for cancer patients–does COVID-19 pandemic change the attitude towards use of telemedicine services?
Source: PLoS One. 2023 Feb 10;18(2):e0280723. doi: 10.1371/journal.pone.0280723 (PMC9917238; doi:10.1371/journal.pone.0280723)
Supplement: S5 Table — (PDF) [file pone.0280723.s005.pdf]

		% Online health related information search.						
		**Internet usage for health related topics**	**Healthy lifestyle and nutrition**	**Prevention**	**Present illness and treatment options**	**Medication**	**Patient rights and physician- and hospitalranking**	**Others**
		Total:<br>N = 271<br>Yes: n = 137	Total:<br>N = 271<br>Yes: n = 120	Total:<br>N = 271<br>Yes: n = 98	Total:<br>N = 271<br>Yes: n = 171	Total:<br>N = 271<br>Yes: n = 124	Total:<br>N = 271<br>Yes: n = 61	Total:<br>N = 271<br>Yes: n = 9
**Gender**	Female<br>Male	64 (50,8)<br>73 (52,1)<br>*(p = 0,826)*	64 (50,8)<br>55 (39,3)<br>*(p = 0,059)*	53 (42,1)<br>45 (32,1)<br>*(p = 0,094)*	86 (68,3)<br>85 (60,7)<br>*(p = 0,200)*	65 (51,6)<br>59 (42,1)<br>*(p = 0,123)*	33 (26,2)<br>28 (20,0)<br>*(p = 0,230)*	5 (4,0)<br>3 (2,1)<br>*(p = 0,384)*
**Age**	≤ 54<br>≥ 55	44 (65,7)<br>93 (47,4)<br>***(p = 0,010)***	38 (56,7)<br>80 (40,8)<br>***(p = 0,024)***	33 (49,3)<br>64 (32,7)<br>***(p = 0,015)***	53 (79,1)<br>117 (59,7)<br>***(p = 0,004)***	39 (58,2)<br>85 (43,4)<br>***(p = 0,036)***	17 (25,4)<br>44 (22,4)<br>*(p = 0,624)*	1 (1,5)<br>7 (3,6)<br>*(p = 0,392)*
**Community size (Inhabitants)**	>= 30.000<br>> 30.000	68 (50,0)<br>68 (55,3)<br>*(p = 0,395)*	59 (43,4)<br>57 (46,3)<br>*(p = 0,632)*	47 (34,6)<br>48 (39,0)<br>*(p = 0,456)*	81 (59,6)<br>84 (68,3)<br>*(p = 0,144)*	54 (39,7)<br>65 (52,8)<br>*(p = 0,034)*	25 (18.4)<br>36 (29,3)<br>*(p = 0,039)*	5 (3,7)<br>3 (2,4)<br>*(p = 0,565)*
**Proximity to university hospital**	≤ 20 km<br>≥ 21 km	71 (53,4)<br>66 (50,4)<br>(p = 0,626)	56 (42,1)<br>63 (48,1)<br>(p = 0,328)	46 (34,6)<br>52 (39,7)<br>(p = 0,390)	86 (64,7)<br>84 (64,1)<br>(p = 0,927)	68 (51,1)<br>55 (42,0)<br>(p = 0,136)	25 (18,8)<br>36 (27,5)<br>(p = 0,094)	3 (2,3)<br>5 (3,8)<br>(p = 0,459)
**Travel time to hospital**	≤ 30 min<br>≥ 31 min	77 (54,6)<br>59 (48,8)<br>(p = 0,345)	66 (46,8)<br>52 (53,0)<br>(p = 0,534)	55 (39,0)<br>43 (35,5)<br>(p = 0,563)	95 (67,4)<br>74 (61,2)<br>(p = 0,294)	74 (52,5)<br>49 (40,5)<br>(p = 0,053)	29 (20,6)<br>32 (26,4)<br>(p = 0,262)	5 (3,5)<br>3 (2,5)<br>(p = 0,617)
**Educational level**	Low<br>Middle + high	27 (33,3)<br>106 (58,9)<br>***(p < 0,001)***	23 (28,4)<br>94 (52,2)<br>***(p < 0,001)***	19 (23,5)<br>77 (42,8)<br>***(p = 0,003)***	36 (44,4)<br>130 (72,2)<br>***(p < 0,001)***	27 (33,3)<br>93 (51,7)<br>***(p = 0,006)***	6 (7,4)<br>52 (28,9)<br>***(p < 0,001)***	4 (4,9)<br>4 (2,2)<br>*(p = 0,239)*
**Occupational level**	Low<br>Middle + high	10 (38,5)<br>124 (52,5)<br>***(p = 0,173)***	5 (19,2)<br>117 (47,5)<br>***(p = 0,006)***	6 (23,1)<br>90 (38,1)<br>*(p = 0.130)*	13 (50,0)<br>154 (65,3)<br>***(p = 0,125)***	11 (42,3)<br>110 (46,6)<br>***(p = 0,676)***	3 (11,5)<br>56 (23,7)<br>*(p = 0,158)*	1 (3,8)<br>7 (3,0)<br>*(p = 0,804)*
**Employed**	No<br>Yes	93 (47,4)<br>42 (62,7)<br>***(p = 0,031)***	75 (38,3)<br>42 (62,7)<br>***(p = 0,001)***	64 (32,7)<br>33 (49,3)<br>***(p = 0,015)***	121 (61,7)<br>47 (70,1)<br>*(p = 0,216)*	84 (42,9)<br>38 (56,7)<br>*(p = 0,050)*	45 (23,0)<br>15 (22,4)<br>*(p = 0,923)*	6 (3,1)<br>2 (3,0)<br>*(p = 0,975)*
**Full time or part time job**	≤ 50%<br>> 50 %	13 (52,0)<br>28 (63,6)<br>*(p = 0,344)*	15 (60,0)<br>26 (59,1)<br>*(p = 0,941)*	11 (44,0)<br>21 (47,7)<br>*(p = 0,765)*	15 (60,0)<br>31 (70,5)<br>*(p = 0,376)*	10 (40,0)<br>27 (61,4)<br>*(p = 0,087)*	4 (16,0)<br>11 (25,0)<br>*(p = 0,384)*	2 (8,0)<br>0 (0,0)<br>*(p = 0,057)*
**Frequency of medical consultation in the last year**	≤ 5 times<br>> 5 times	18 (41,9)<br>117 (53.7)<br>*(p = 0,157)*	16 (37,2)<br>101 (46,3)<br>*(p = 0,272)*	11 (25,6)<br>85 (39,0)<br>***(p = 0,096)***	21 (48,8)<br>148 (67,9)<br>*(p = 0,017)*	14 (32,6)<br>107 (49,1)<br>*(p = 0,047)*	7 (16,3)<br>53 (24,3)<br>*(p = 0,253)*	1 (2,3)<br>8 (3,7)<br>*(p = 0,659)*
**Missed appointments in the past**	No<br>Yes	121 (50,8)<br>14 (51,9)<br>*(p = 0,921)*	106 (44,5)<br>12 (44,4)<br>*(p = 0,993)*	85 (35,7)<br>12 (44,4)<br>*(p = 0,372)*	153 (64,3)<br>16 (59,3)<br>*(p = 0,607)*	112 (47,1)<br>10 (37,0)<br>*(p = 0,322)*	56 (23,5)<br>5 (18,5)<br>*(p = 0,558)*	9 (3,8)<br>0 (0,0)<br>*(p = 0,304)*
**Insurance status**	Statutory health insurance<br>Private health insurance	87 (47,8)<br>49 (59,0)<br>*(p = 0,090)*	73 (40,1)<br>45 (54,2)<br>***(p = 0,032)***	60 (33,0)<br>38 (45,8)<br>***(p = 0,045)***	107 (58,8)<br>63 (75,9)<br>***(p = 0,007)***	80 (44,0)<br>44 (53,0)<br>*(p = 0,171)*	35 (19,2)<br>25 (30,1)<br>***(p = 0,049)***	7 (3,8)<br>1 (1,2)<br>*(p = 0,244)*
**Knowledge of the definition of eHealth**	No<br>Yes	87 (43,3)<br>50 (74,6)<br>***(p < 0,001)***	76 (37,8)<br>44 (65,7)<br>***(p < 0,001)***	57 (28,4)<br>41 (61,2)<br>***(p < 0,001)***	115 (57,2)<br>56 (83,6)<br>***(p < 0,001)***	82 (40,8)<br>42 (62,7)<br>***(p = 0,002)***	34 (16,9)<br>27 (40,3)<br>***(p < 0,001)***	6 (3,0)<br>3 (4,5)<br>*(p = 0,557)*
**Medication intake**	≤ 5 different medication/day<br>≥ 6 different medication/day	88 (53,3)<br>47 (47,0)<br>*(p = 0,317)*	77 (46,7)<br>41 (41,0)<br>*(p = 0,368)*	57 (34,5)<br>39 (39,0)<br>*(p = 0,465)*	105 (63,6)<br>64 (64,0)<br>*(p = 0,952)*	78 (47,3)<br>44 (44,0)<br>*(p = 0,604)*	38 (23,0)<br>23 (23,0)<br>*(p = 0,995)*	6 (3,6)<br>3 (3,0)<br>*(p = 0,782)*
**Participation before COVID-19**	Yes<br>No	38 (52,8)<br>99 (49,7)<br>(p = 0,660)	28 (38,9)<br>92 (46,2)<br>(p = 0,282)	23 (31,9)<br>75 (37,7)<br>(p = 0,385)	44 (61,1)<br>127 (63,8)<br>(p = 0,683)	26 (36,1)<br>98 (49,2)<br>(p = 0,055)	16 (22,2)<br>45 (22,6)<br>(p = 0,946)	1 (1,4)<br>8 (4,0)<br>(p = 0,286)
**Reasons for medical consultation**	Active therapy<br>Follow up care	114 (51,8)<br>22 (52,4)<br>(p = 0,947)	95 (43,2)<br>24 (57,1)<br>(p = 0,096)	83 (37,7)<br>14 (33,3)<br>(p = 0,589)	142 (64,5)<br>28 (66,7)<br>(p = 0,792)	101 (45,9)<br>22 (52,4)<br>(p = 0,441)	49 (22,3)<br>11 (26,2)<br>(p = 0,580)	9 (4,1)<br>0 (0,0)<br>(p = 0,182)
**Type of cancer**	Solid<br>Hematological	59 /46,5)<br>68 (59,1)<br>(p = 0,049)	57 (44,9)<br>56 (48,7)<br>(p = 0,553)	46 (36,2)<br>46 (40,0)<br>(p = 0,545)	82 (64,6)<br>82 (71,3)<br>(p = 0,263)	61 (48,0)<br>56 (48,7)<br>(p = 0,918)	25 (19,7)<br>32 (27,8)<br>(p = 0,136)	7 (5,5)<br>2 (1,7)<br>(p = 0,121)

S5 Table. Online health related information search.
