# Supplementary material for: The potential of eHealth for cancer patients–does COVID-19 pandemic change the attitude towards use of telemedicine services?
Source: PLoS One. 2023 Feb 10;18(2):e0280723. doi: 10.1371/journal.pone.0280723 (PMC9917238; doi:10.1371/journal.pone.0280723)
Supplement: S6 Table — (PDF) [file pone.0280723.s006.pdf]

		% of patients obtain health information from the following sources							
		**Social Media**	**Blogs**	**Discussion forums**	**HP of self-help groups**	**HP hospitals/physicians**	**HP medical societies**	**HP pharmaceutical companies**	**Others**
		Total:<br>N = 268<br>Ja: n = 33	Total:<br>N = 268<br>Ja: n = 23	Total:<br>N = 268<br>Ja: n = 45	Total:<br>N = 268<br>Ja: n = 48	Total:<br>N = 268<br>Ja: n = 127	Total:<br>N = 268<br>Ja: n = 127	Total:<br>N = 268<br>Ja: n = 55	Total:<br>N = 268<br>Ja: n = 14
**Gender**	Female<br>Male	16 (12,9)<br>17 (12,1)<br>*(p = 0,852)*	7 (5,6)<br>16 (11,4)<br>*(p = 0,096)*	21 (16,9)<br>23 (16,4)<br>*(p = 0,912)*	29 (23,4)<br>18 (12,9)<br>***(p = 0,026)***	68 (54,8)<br>59 (42,1)<br>***(p = 0,039)***	61 (49,2)<br>66 (47,1)<br>*(p = 0,739)*	29 (23,4)<br>26 (18,6)<br>*(p = 0,336)*	6 (4,8)<br>8 (5,7)<br>*(p = 0,751)*
**Age**	≤ 54<br>≥ 55	10 (14,9)<br>23 (11,9)<br>*(p = 0,515)*	13 (19,4)<br>10 (5,2)<br>***(p < 0,001)***	22 (32,8)<br>21 (10,4)<br>***(p < 0,001)***	14 (20,9)<br>33 (17,0)<br>*(p = 0,476)*	36 (53,7)<br>90 (46,4)<br>*(p = 0,300)*	46 (68,7)<br>80 (41,2)<br>***(p < 0,001)***	24 (35,8)<br>31 (16,0)<br>***(p = 0,001)***	5 (7,5)<br>9 (4,6)<br>*(p = 0,376)*
**Community size (Inhabitants)**	>= 30.000<br>> 30.000	18 (13,4)<br>12 (9,8)<br>*(p = 0,359)*	7 (5,2)<br>16 (13,0)<br>*(p = 0,029)*	20 (14,9)<br>23 (18,7)<br>*(p = 0,418)*	22 (16,4)<br>24 (19,5)<br>*(p = 0,518)*	57 (42,5)<br>65 (52,8)<br>*(p = 0,098)*	59 (44,0)<br>65 (52,8)<br>*(p = 0,158)*	25 (18,7)<br>29 (23,6)<br>*(p = 0,333)*	5 (3,7)<br>9 (7,3)<br>*(p = 0,206)*
**Proximity to university hospital**	≤ 20 km<br>≥ 21 km	13 (9,8)<br>19 (14,6)<br>(p = 0,230)	13 (9,8)<br>10 (7,7)<br>(p = 0,550)	22 (16,5)<br>22 (16,9)<br>(p = 0,934)	21 (15,8)<br>26 (20,0)<br>(p = 0,373)	63 (47,4)<br>63 (48,5)<br>(p = 0,859)	62 (46,6)<br>65 (50,0)<br>(p = 0,583)	28 (21,1)<br>27 (20,8)<br>(p = 0,955)	9 (6,8)<br>5 (3,8)<br>(p = 0,291)
**Travel time to hospital**	≤ 30 min<br>≥ 31 min	15 (10,8)<br>16 (13,2)<br>(p = 0,546)	13 (9,4)<br>9 (7,4)<br>(p = 0,580)	29 (20,9)<br>15 (12,4)<br>(p = 0,069)	23 (16,5)<br>24 (19,8)<br>(p = 0,492)	69 (49,6)<br>57 (47,1)<br>(p = 0,684)	70 (50,4)<br>57 (47,1)<br>(p = 0,601)	33 (23,7)<br>22 (18,2)<br>(p = 0,274)	8 (5,8)<br>6 (5,0)<br>(p = 0,776)
**Educational level**	Low<br>Middle + high	11 (13,9)<br>20 (11,1)<br>*(p = 0,521)*	3 (3,8)<br>19 (10,6)<br>*(p = 0,072)*	5 (6,3)<br>38 (21,1)<br>***(p = 0,003)***	11 (13,9)<br>36 (20,0)<br>*(p = 0,243)*	26 (32,9)<br>97 (53,9)<br>***(p = 0,002)***	19 (24,1)<br>107 (59,4)<br>***(p < 0,001)***	8 (10,1)<br>47 (26,1)<br>***(p = 0,004)***	2 (2,5)<br>12 (6,7)<br>***(p = 0,175)***
**Occupational level**	Low<br>Middle + high	5 (20,0)<br>26 (11,1)<br>*(p = 0,190)*	0 (0,0)<br>22 (9,4)<br>***(p = 0,110)***	2 (8,0)<br>42 (17,9)<br>*(p = 0,211)*	1 (4,0)<br>46 (19,6)<br>*(p = 0,054)*	7 (28,0)<br>117 (49,8)<br>*(p=0,038)*	9 (36,0)<br>117 (49,8)<br>***(p = 0,190)***	2 (8,0)<br>53 (22,6)<br>***(p = 0,090)***	1 (4,0)<br>13 (5.5)<br>***(p = 0,747)***
**Employed**	No<br>Yes	26 (13,4)<br>5 (7,5)<br>*(p = 0,195)*	16 (8,2)<br>6 (9,0)<br>*(p = 0,857)*	28 (14,4)<br>16 (23,9)<br>*(p = 0,075)*	32 (16,5)<br>15 (22,4)<br>*(p = 0,279)*	87 (44,8)<br>38 (56,7)<br>*(p = 0,094)*	79 (40,7)<br>48 (71,6)<br>***(p < 0,001)***	34 (17,5)<br>21 (31,3)<br>***(p = 0,017)***	9 (4,6)<br>5 (7,5)<br>*(p = 0,376)*
**Full time or part time job**	≤ 50%<br>> 50 %	0 (0,0)<br>5 (11,4)<br>*(p = 0,080)*	1 (4,0)<br>5 (11,4)<br>*(p = 0,297)*	5 (20,0)<br>11 (25,0)<br>*(p = 0,636)*	6 (24,0)<br>10 (22,7)<br>*(p = 0,904)*	12 (48,0)<br>25 (56,8)<br>*(p = 0,480)*	14 (56,0)<br>33 (75,0)<br>*(p = 0,104)*	8 (32,0)<br>12 (27,3)<br>*(p = 0,677)*	2 (8,0)<br>3 (6,8)<br>*(p = 0,856)*
**Frequency of medical consultation in the last year**	≤ 5 times<br>> 5 times	6 (14,0)<br>27 (12,4)<br>*(p = 0,777)*	3 (7,0)<br>20 (9,2)<br>*(p = 0,642)*	2 (4,7)<br>43 (19,7)<br>***(p = 0,017)***	5 (11,6)<br>43 (19,7)<br>*(p = 0,210)*	20 (46,5)<br>106 (48,6)<br>*(p = 0,800)*	12 (27,9)<br>114 (52,3)<br>***(p = 0,003)***	4 (9,3)<br>50 (22,9)<br>*(p = 0,044)*	1 (2,3)<br>13 (6,0)<br>*(p = 0,333)*
**Missed appointments in the past**	No<br>Yes	28 (11,8)<br>5 (18,5)<br>*(p = 0,318)*	17 (7,2)<br>6 (22,2)<br>***(p = 0,009)***	39 (16,5)<br>6 (22,2)<br>*(p = 0,450)*	44 (18,6)<br>4 (14,8)<br>*(p = 0,632)*	114 (48,1)<br>12 (44,4)<br>*(p = 0,719)*	114 (48,1)<br>11 (40,7)<br>*(p = 0,468)*	50 (21,1)<br>5 (18,5)<br>*(p = 0,755)*	12 (5,1)<br>2 (7,4)<br>*(p = 0,607)*
**Insurance status**	Statutory health insurance<br>Private health insurance	27 (14,9)<br>5 (6,1)<br>***(p = 0,043)***	13 (7,2)<br>9 (11,0)<br>*(p = 0,303)*	28 (15,5)<br>16 (19,5)<br>*(p = 0,416)*	28 (15,5)<br>19 (23,2)<br>*(p = 0,131)*	78 (43,1)<br>48 (58,5)<br>***(p = 0,020)***	78 (43,1)<br>49 (59,8)<br>***(p = 0,012)***	33 (18,2)<br>22 (26,8)<br>*(p = 0,112)*	11 (6,1)<br>3 (3,7)<br>*(p = 0,418)*
**Knowledge of the definition of eHealth**	No<br>Yes	20 (10,0)<br>13 (19,4)<br>***(p = 0,043)***	14 (7,0)<br>9 (13,4)<br>*(p = 0,104)*	25 (12,5)<br>20 (29,9)<br>***(p = 0,001)***	30 (15,0)<br>18 (26,9)<br>***(p = 0,029)***	81 (40,5)<br>46 (68,7)<br>***(p < 0,001)***	78 (39,0)<br>49 (73,1)<br>***(p < 0,001)***	32 (16,0)<br>23 (34,3)<br>***(p = 0,001)***	10 (5,0)<br>4 (6,0)<br>*(p = 0,758)*
**Medication intake**	≤ 5 different medication/day<br>≥ 6 different medication/day	21 (12,8)<br>12 (12,0)<br>*(p = 0,848)*	17 (10,4)<br>6 (6,0)<br>*(p = 0,222)*	27 (16,5)<br>18 (18,0)<br>*(p = 0,747)*	25 (15,2)<br>23 (23,0)<br>*(p = 0,113)*	80 (48,8)<br>45 (45,0)<br>*(p = 0,551)*	79 (48,2)<br>47 (47,0)<br>*(p = 0,853)*	36 (22,0)<br>18 (18,0)<br>*(p = 0,440)*	7 (4,3)<br>7 (7,0)<br>*(p = 0,337)*
**Participation before COVID-19**	Yes<br>No	4 (5,6)<br>29 (14,8)<br>(p = 0,041)	7 (9,7)<br>16 (8,2)<br>(p = 0,686)	13 (18,1)<br>32 (16,3)<br>(p = 0,737)	12 (16,7)<br>36 (18,4)<br>(p = 0,748)	31 (43,1)<br>96 (49,0)<br>(p = 0,389)	33 (45,8)<br>94 (48,0)<br>(p = 0,757)	12 (16,7)<br>43 (21,9)<br>(p = 0,343)	6 (8,3)<br>8 (4,1)<br>(p = 0,166)
**Reasons for medical consultation**	Active therapy<br>Follow up care	24 (11,0)<br>9 (21,4)<br>(p = 0,061)	20 (9,1)<br>3 (7,1)<br>(p = 0,677)	41 (18,7)<br>4 (9,5)<br>(p = 0,148)	43 (19,6)<br>5 (11,9)<br>(p = 0,236)	99 (45,2)<br>27 (64,3)<br>(p = 0,023)	107 (48,9)<br>20 (47,6)<br>(p = 0,883)	46 (21,0)<br>9 (21,4)<br>(p = 0,951)	12 (5,5)<br>2 (4,8)<br>(p = 0,850)
**Type of cancer**	Solid<br>Hematological	15 (11,9)<br>16 (13,9)<br>(p = 0,642)	10 (7,9)<br>12 (10,4)<br>(p = 0,501)	19 (15,1)<br>24 (20,9)<br>(p = 0,241)	22 (17,5)<br>26 (22,6)<br>(p = 0,318)	63 (50,0)<br>56 (48,7)<br>(p = 0,840)	57 (45,2)<br>62 (53,9)<br>(p = 0,178)	24 (19,0)<br>25 (21,7)<br>(p = 0,604)	6 (4,8)<br>8 (7,0)<br>(p = 0,467)

S6 Table. Sources of online health information.
