# Supplementary material for: The potential of eHealth for cancer patients–does COVID-19 pandemic change the attitude towards use of telemedicine services?
Source: PLoS One. 2023 Feb 10;18(2):e0280723. doi: 10.1371/journal.pone.0280723 (PMC9917238; doi:10.1371/journal.pone.0280723)
Supplement: S7 Table — (PDF) [file pone.0280723.s007.pdf]

| | | % Online search for health information by patients, "know how" positive. | | | | | | | |
|---|---|---|---|---|---|---|---|---|---|
| | | **I know how to find helpful information on the internet.** | **I know how to use the internet to answer my questions about health.** | **I know which health resources are available on the internet.** | **I know where to find helpful health resources on the internet.** | **I know how to use the health information to help me.** | **I have the skills I need to evaluate the health resources I find on the internet.** | **I can tell high quality resources from low quality health resources.** | **I feel confident in using information from the internet to make health decisions.** |
| | | Total:<br>N = 266<br>Yes: n = 163<br>No: n = 53<br>I.d.k.: n = 50 | Total:<br>N = 267<br>Yes: n = 162<br>No: n = 61<br>I.d.k.: n = 44 | Total:<br>N = 267<br>Yes: n = 139<br>No: n = 78<br>I.d.k.: n = 50 | Total:<br>N = 266<br>Yes: n = 151<br>No n = 64<br>I.d.k.: n = 51 | Total:<br>N = 267<br>Yes: n = 148<br>No: n = 67<br>I.d.k. n = 52 | Total:<br>N = 267<br>Yes: n = 181<br>No: n = 38<br>I.d.k. n = 48 | Total:<br>N = 267<br>Yes: n = 144<br>No: n = 68<br>I.d.k.: n = 55 | Total:<br>N = 267<br>Yes n = 74<br>No: n = 137<br>I.d.k.: n = 56 |
| **Gender** | Female<br>Male | 80 (64,5)<br>82 (58,6)<br>*(p = 0,612)* | 79 (63,2)<br>82 (58,6)<br>*(p = 0,684)* | 71 (56,8)<br>68 (48,6)<br>*(p = 0,382)* | 75 (60,5)<br>75 (53,6)<br>*(p = 0,450)* | 73 (58,4)<br>74 (52,9)<br>*(p = 0,570)* | 92 (73,6)<br>88 (62,9)<br>***(p = 0,045)*** | 70 (56,0)<br>73 (52,1)<br>*(p = 0,684)* | 37 (29,6)<br>37 (26,4)<br>*(p = 0,736)* |
| **Age** | ≤ 54<br>≥ 55 | 51 (76,1)<br>110 (56,7)<br>***(p = 0,015)*** | 48 (71,6)<br>112 (57,4)<br>*(p = 0,090)* | 43 (64,2)<br>95 (48,7)<br>***(p = 0,039)*** | 48 (71,6)<br>101 (52,1)<br>***(p = 0,008)*** | 53 (79,1)<br>94 (48,2)<br>***(p < 0,001)*** | 54 (80,6)<br>126 (64,6)<br>***(p = 0,029)*** | 48 (71,6)<br>95 (48,7)<br>***(p = 0,003)*** | 21 (31,3)<br>53 (27,2)<br>*(p = 0,147)* |
| **Community size (Inhabitants)** | >= 30.000<br>> 30.000 | 80 (59,3)<br>76 (62,3)<br>*(p = 0,369)* | 80 (58,8)<br>75 (61,5)<br>*(p = 0,737)* | 67 (49,3)<br>65 (53,3)<br>*(p = 0,776)* | 74 (54,8)<br>69 (56,6)<br>*(p = 0,772)* | 70 (51,5)<br>72 (59,0)<br>*(p = 0,256)* | 89 (65,4)<br>85 (69,7)<br>*(p = 0,733)* | 67 (49,3)<br>70 (57,4)<br>*(p = 0,388)* | 41 (30,1)<br>30 (24,6)<br>*(p = 0,535)* |
| **Proximity to university hospital** | ≤ 20 km<br>≥ 21 km | 82 (62,1)<br>79 (60,3)<br>(p = 0,955) | 82 (62,1)<br>78 (59,1)<br>(p = 0,705) | 71 (53,8)<br>67 (50,8)<br>(p = 0,881) | 79 (59,8)<br>70 (53,4)<br>(p = 0,554) | 77 (58,3)<br>69 (52,3)<br>(p = 0,588) | 87 (65,9)<br>92 (69,7)<br>(p = 0,748) | 72 (54,5)<br>70 (53,0)<br>(p = 0,957) | 37 (28,0)<br>36 (27,3)<br>(p = 0,970) |
| **Travel time to hospital** | ≤ 30 min<br>≥ 31 min | 89 (64,0)<br>72 (59,0)<br>(p = 0,343) | 88 (62,9)<br>72 (59,0)<br>(p = 0,794) | 79 (56,4)<br>59 (48,49<br>(p = 0,416) | 85 (61,2)<br>64 (52,5)<br>(p = 0,320) | 84 (60,0)<br>61 (50,0)<br>(p = 0,252) | 97 (69,3)<br>81 (66,4)<br>(p = 0,865) | 81 (57,9)<br>61 (50,0)<br>(p = 0,444) | 43 (30,7)<br>29 (23,8)<br>(p = 0,450) |
| **Educational level** | Low<br>Middle + high | 33 (41,8)<br>127 (70,6)<br>***(p < 0,001)*** | 33 (41,3)<br>126 (70,0)<br>***(p < 0,001)*** | 27 (33,8)<br>110 (61,1)<br>***(p < 0,001)*** | 28 (35,4)<br>120 (66,7)<br>***(p < 0,001)*** | 31 (38,8)<br>112 (62,2)<br>***(p = 0,001)*** | 42 (52,5)<br>133 (73,9)<br>***(p = 0,003)*** | 29 (36,3)<br>110 (61,1)<br>***(p = 0,001)*** | 16 (20,0)<br>54 (30,0)<br>*(p = 0,074)* |
| **Occupational level** | Low<br>Middle + high | 8 (32,0)<br>152 (64,7)<br>***(p < 0,001)*** | 9 (34,6)<br>150 (63,8)<br>***(p < 0,001)*** | 8 (30,8)<br>129 (54,9)<br>***(p = 0,030)*** | 9 (36,0)<br>139 (59,1)<br>***(p = 0,003)*** | 9 (34,6)<br>153 (57,4)<br>***(p = 0,052)*** | 12 (46,2)<br>164 (69,8)<br>***(p = 0,013)*** | 6 (23,1)<br>134 (57,0)<br>***(p = 0,002)*** | 7 (26,9)<br>64 (27,2)<br>*(p = 0,725)* |
| **Employed** | No<br>Yes | 109 (56,2)<br>51 (76,1)<br>***(p = 0,015)*** | 111 (56,9)<br>48 (71,6)<br>*(p = 0,104)* | 88 (45,1)<br>49 (73,1)<br>***(p < 0,001)*** | 101 (52,1)<br>47 (70,1)<br>***(p = 0,026)*** | 93 (47,7)<br>51 (76,1)<br>***(p < 0,001)*** | 123 (63,1)<br>54 (80,6)<br>***(p = 0,017)*** | 96 (49,2)<br>45 (67,2)<br>***(p = 0,037)*** | 45 (23,1)<br>26 (38,8)<br>***(p = 0,044)*** |
| **Full time or part time job** | ≤ 50%<br>> 50 % | 16 (64,0)<br>34 (77,3)<br>*(p = 0,370)* | 15 (60,0)<br>32 (72,7)<br>*(p = 0,340)* | 16 (64,0)<br>32 (72,7)<br>*(p = 0,726)* | 15 (60,0)<br>31 (70,5)<br>*(p = 0,615)* | 18 (72,0)<br>32 (72,7)<br>*(p = 0,915)* | 18 (72,0)<br>35 (79,5)<br>*(p = 0,715)* | 16 (64,0)<br>28 (63,6)<br>*(p = 0,684)* | 12 (48,0)<br>14 (31,8)<br>*(p = 0,373)* |
| **Frequency of medical consultation in the last year** | ≤ 5 times<br>> 5 times | 18 (41,9)<br>142 (64,8)<br>***(p = 0,001)*** | 18 (41,9)<br>141 (64,4)<br>***(p = 0,007)*** | 14 (32,6)<br>122 (55,7)<br>***(p = 0,001)*** | 15 (34,9)<br>133 (60,7)<br>***(p < 0,001)*** | 14 (32,6)<br>131 (59,8)<br>***(p < 0,001)*** | 17 (39,5)<br>161 (73,5)<br>***(p < 0,001)*** | 13 (30,2)<br>128 (58,4)<br>***(p = 0,001)*** | 8 (18,6)<br>65 (29,7)<br>*(p = 0,031)* |
| **Missed appointments in the past** | No<br>Yes | 142 (60,2)<br>19 (70,4)<br>*(p = 0,459)* | 142 (59,9)<br>18 (66,7)<br>*(p = 0,394)* | 127 (53,6)<br>11 (40,7)<br>*(p = 0,059)* | 135 (57,2)<br>15 (55,6)<br>*(p = 0,669)* | 128 (54,0)<br>17 (63,0)<br>*(p = 0,658)* | 157 (66,2)<br>21 (77,8)<br>*(p = 0,431)* | 128 (54,0)<br>14 (51,9)<br>*(p = 0,524)* | 66 (27,8)<br>6 (22,2)<br>*(p = 0,509)* |
| **Insurance status** | Statutory health insurance<br>Private health insurance | 99 (54,7)<br>62 (75,6)<br>***(p < 0,001)*** | 99 (54,7)<br>61 (73,5)<br>***(p=0,011)*** | 86 (47,5)<br>53 (63,9)<br>***(p = 0,032)*** | 92 (50,8)<br>58 (70,7)<br>***(p = 0,007)*** | 92 (50,8)<br>54 (65,1)<br>*(p = 0,082)* | 119 (65,7)<br>60 (72,3)<br>*(p = 0,567)* | 91 (50,3)<br>51 (61,4)<br>*(p = 0,227)* | 49 (27,1)<br>25 (30,1)<br>*(p = 0,845)* |
| **Knowledge of the definition of eHealth** | No<br>Yes | 105 (52,8)<br>58 (86,6)<br>***(p < 0,001)*** | 109 (54,5)<br>53 (79,1)<br>***(p < 0,001)*** | 90 (45,0)<br>49 (73,1)<br>***(p < 0,001)*** | 101 (50,8)<br>50 (74,6)<br>***(p < 0,001)*** | 96 (48,0)<br>52 (77,6)<br>***(p < 0,001)*** | 118 (59,0)<br>63 (94,0)<br>***(p < 0,001)*** | 91 (45,5)<br>53 (79,1)<br>***(p < 0,001)*** | 51 (25,5)<br>23 (34,3)<br>***(p = 0,007)*** |
| **Medication intake** | ≤ 5 different medication/day<br>≥ 6 different medication/day | 102 (62,2)<br>60 (60,6)<br>*(p = 0,672)* | 101 (61,2)<br>59 (59,6)<br>*(p = 0,351)* | 90 (54,5)<br>48 (48,5)<br>*(p = 0,310)* | 95 (57,9)<br>55 (55,6)<br>*(p = 0,359)* | 96 (58,2)<br>51 (51,5)<br>*(p = 0,284)* | 112 (67,9)<br>67 (67,7)<br>*(p = 0,152)* | 88 (53,3)<br>55 (55,6)<br>*(p = 0,631)* | 48 (29,1)<br>26 (26,3)<br>*(p = 0,668)* |
| **Participation before COVID-19** | Yes<br>No | 42 (57,5)<br>121 (62,7)<br>(p = 0,512) | 40 (54,8)<br>122 (62,9)<br>(p = 0,451) | 34 (46,6)<br>105 (54,1)<br>(p = 0,230) | 38 (52,1)<br>113 (58,5)<br>(p = 0,517) | 34 (46,6)<br>114 (58,8)<br>(p = 0,142) | 44 (60,3)<br>137 (70,6)<br>(p = 0,184) | 34 (46,6)<br>110 (56,7)<br>(p = 0,195) | 16 (21,9)<br>58 (29,9)<br>(p = 0,376) |
| **Reasons for medical consultation** | Active therapy<br>Follow up care | 134 (61,5)<br>27 (64,3)<br>(p = 0,027) | 134 (61,2)<br>26 (61,9)<br>(p = 0,154) | 113 (51,6)<br>24 (57,1)<br>(p = 0,404) | 123 (56,4)<br>26 (61,9)<br>(p = 0,071) | 123 (56,2)<br>23 (54,8)<br>(p = 0,559) | 150 (68,5)<br>29 (69,0)<br>(p = 0,223) | 119 (54,3)<br>23 (54,8)<br>(p = 0,806) | 62 (28,3)<br>10 (23,8)<br>(p = 0,087) |
| **Type of cancer** | Solid<br>Hematological | 77 (61,1)<br>78 (67,8)<br>(p = 0,551) | 79 (62,2)<br>76 (66,1)<br>(p = 0,811) | 70 (55,1)<br>65 (56,5)<br>(p = 0,974) | 72 (57,1)<br>72 (62,6)<br>(p = 0,647) | 66 (52,0)<br>74 (64,3)<br>(p = 0,111) | 86 (67,7)<br>85 (73,9)<br>(p = 0,517) | 66 (52,0)<br>73 (63,5)<br>(p = 0,164) | 31 (24,4)<br>41 (35,7)<br>(p = 0,161) |

S7 Table. Assessment of online searches for health information by patients (eHeals).
