# Supplementary material for: The potential of eHealth for cancer patients–does COVID-19 pandemic change the attitude towards use of telemedicine services?
Source: PLoS One. 2023 Feb 10;18(2):e0280723. doi: 10.1371/journal.pone.0280723 (PMC9917238; doi:10.1371/journal.pone.0280723)
Supplement: S8 Table — (PDF) [file pone.0280723.s008.pdf]

		% Online communication with physicians/hospitals helpful for patients							
		**Online appointment scheduling**	**Online appointment reminder (SMS/E-Mail)**	**Transfer of general information via e-mail**	**Zusendung Untersuchungs - ergebnisse via E-Mail**	**Zusendung Arztbriefe via E-Mail**	**Zusendung Medikations-pläne via E-Mail**	**Online Chat**	**Online video calls**
		Total:<br>N = 262<br>Ja: n = 173<br>Nein: n = 50<br>W.n.: n = 39	Total:<br>N = 262<br>Ja: n = 174<br>Nein: n = 51<br>W.n.: n = 37	Total:<br>N = 263<br>Ja: n = 163<br>Nein: n = 60<br>W.n.: n = 40	Total:<br>N = 264<br>Ja: n = 160<br>Nein: n = 67<br>W.n.: n = 37	Total:<br>N = 263<br>Ja: n = 164<br>Nein: n = 63<br>W.n.: n = 36	Total:<br>N = 263<br>Ja: n = 159<br>Nein: n = 66<br>W.n.: n = 38	Total:<br>N = 263<br>Ja: n = 149<br>Nein: n = 72<br>W.n.: n = 42	Total:<br>N = 262<br>Ja: n = 111<br>Nein: n = 96<br>W.n.: n = 55
**Gender**	Female<br>Male	73 (59,8)<br>100 (72,5)<br>*(p = 0,097)*	76 (62,3)<br>98 (71,0)<br>*(p = 0,327)*	73 (59,3)<br>89 (64,5)<br>*(p = 0,601)*	68 (54,8)<br>91 (65,9)<br>*(p = 0,182)*	66 (53,7)<br>97 (70,3)<br>***(p = 0,021)***	66 (53,7)<br>92 (66,7)<br>*(p = 0,098)*	71 (57,7)<br>77 (55,8)<br>*(p = 0,671)*	53 (43,4)<br>58 (42,0)<br>*(p = 0,919)*
**Age**	≤ 54<br>≥ 55	51 (77,3)<br>120 (62,5)<br>***(p = 0,041)***	54 (81,8)<br>118 (61,5)<br>***(p = 0,003)***	50 (75,8)<br>110 (57,0)<br>***(p = 0,008)***	48 (72,2)<br>110 (56,7)<br>***(p = 0,038)***	50 (75,8)<br>112 (58,0)<br>***(p = 0,015)***	47 (71,2)<br>109 (56,5)<br>***(p = 0,044)***	47 (70,1)<br>99 (51,6)<br>***(p = 0,011)***	38 (57,6)<br>73 (38,0)<br>***(p = 0,010)***
**Community size (Inhabitants)**	>= 30.000<br>> 30.000	83 (62,4)<br>84 (70,0)<br>*(p = 0,200)*	81 (60,9)<br>87 (72,5)<br>*(p = 0,063)*	72 (54,1))<br>86 (71,1)<br>***(p = 0,010)***	74 (55,2)<br>79 (65,3)<br>*(p = 0,196)*	74 (55,6)<br>84 (69,4)<br>*(p = 0,063)*	74 (55,6)<br>78 (64,5)<br>*(p = 0,202)*	63 (47,4)<br>79 (65,3)<br>*(p = 0,016)*	47 (35,3)<br>59 (49,2)<br>*(p = 0,079)*
**Proximity to university hospital**	≤ 20 km<br>≥ 21 km	86 (66,2)<br>86 (66,7)<br>(p = 0,008)	88 (67,7)<br>85 (65,9)<br>(p =0,128)	85 (64,9)<br>77 (59,7)<br>(p = 0,194)	79 (60,3)<br>79 (60,8)<br>(p = 0,225)	81 (61,8)<br>81 (62,8)<br>(p = 0,263)	79 (60,3)<br>78 (60,5)<br>(p = 0,158)	79 (60,3)<br>68 (52,7)<br>(p = 0,249)	55 (42,3)<br>56 (43,4)<br>(p = 0,184)
**Travel time to hospital**	≤ 30 min<br>≥ 31 min	91 (66,4)<br>81 (66,9)<br>(p = 0,041)	94 (68,6)<br>79 (65,3)<br>(p = 0,417)	89 (65,0)<br>72 (59,5)<br>(p = 0,416)	89 (64,5)<br>68 (56,2)<br>(p = 0,394)	88 (64,2)<br>73 (60,3)<br>(p = 0,716)	86 (62,8)<br>70 (57,9)<br>(p = 0,722)	81 (59,1)<br>65 (53,7)<br>(p = 0,631)	61 (44,5)<br>50 (41,3)<br>(p = 0,866)
**Educational level**	Low<br>Middle + high	41 (53,2)<br>129 (72,1)<br>***(p = 0,007)***	40 (51,9)<br>131 (73,2)<br>***(p = 0,002)***	37 (47,4)<br>123 (68,7)<br>***(p = 0,003)***	36 (45,6)<br>120 (67,0)<br>***(p = 0,005)***	35 (44,9)<br>125 (69,8)<br>***(p = 0,001)***	34 (43,6)<br>121 (67,6)<br>***(p = 0,001)***	35 (44,9)<br>110 (61,5)<br>*(p = 0,014)*	19 (24,7)<br>91 (50,8)<br>***(p = 0,001)***
**Occupational level**	Low<br>Middle + high	12 (50,0)<br>159 (68,2)<br>*(p = 0,076)*	11 (45,8)<br>161 (69,1)<br>***(p = 0,009)***	11 (45,8)<br>150 (64,1)<br>*(p = 0,072)*	8 (32,0)<br>149 (63,7)<br>***(p = 0,003)***	7 (29,2)<br>154 (65,8)<br>***(p = 0,001)***	10 (41,7)<br>146 (62,4)<br>***(p = 0,048)***	9 (37,5)<br>137 (58,5)<br>***(p = 0,007)***	5 (20,8)<br>105 (45,1)<br>***(p = 0,001)***
**Employed**	No<br>Yes	120 (62,2)<br>52 (80,0)<br>***(p = 0,030)***	117 (60,6)<br>56 (86,2)<br>***(p < 0,001)***	109 (56,2)<br>53 (81,5)<br>***(p < 0,001)***	111 (56,9)<br>47 (72,3)<br>***(p = 0,028)***	112 (57,7)<br>50 (76,9)<br>***(p = 0,006)***	113 (58,2)<br>44 (67,7)<br>*(p = 0,107)*	98 (50,8)<br>49 (74,2)<br>***(p = 0,004)***	73 (37,8)<br>38 (58,5)<br>***(p = 0,014)***
**Full time or part time job**	≤ 50%<br>> 50 %	15 (65,2)<br>37 (84,1)<br>*(p = 0,207)*	18 (78,3)<br>37 (84,1)<br>*(p = 0,766)*	18 (78,3)<br>34 (77,3)<br>*(p = 0,712)*	15 (65,2)<br>32 (72,7)<br>*(p = 0,788)*	15 (65,2)<br>35 (79,5)<br>*(p = 0,379)*	14 (60,9)<br>30 (68,2)<br>*(p = 0,498)*	18 (75,0)<br>31 (70,5)<br>*(p = 0,447)*	11 (47,8)<br>27 (61,4)<br>*(p = 0,472)*
**Frequency of medical consultation in the last year**	≤ 5 times<br>> 5 times	22 (52,4)<br>150 (69,1)<br>*(p = 0,083)*	24 (57,1)<br>148 (68,2<br>*(p = 0,279)*	18 (42,9)<br>142 (65,4)<br>*(p = 0,022)*	17 (40,5)<br>138 (63,6)<br>***(p = 0,017)***	18 (42,9)<br>142 (65,4)<br>*(p = 0,022)*	19 (45,2)<br>136 (62,7)<br>***(p = 0,102)***	16 (38,1)<br>130 (59,9)<br>*(p = 0,033)*	10 (23,8)<br>100 (46,1)<br>***(p = 0,028)***
**Missed appointments in the past**	No<br>Yes	152 (65,5)<br>18 (66,7)<br>*(p = 0,353)*	149 (64,2)<br>22 (81,5)<br>*(p = 0,164)*	143 (61,4)<br>17 (63,0)<br>*(p = 0,987)*	138 (59,0)<br>20 (74,1)<br>*(p = 0,299)*	142 (60,9)<br>20 (74,1)<br>*(p = 0,381)*	139 (59,7)<br>18 (66,7)<br>*(p = 0,762)*	134 (57,5)<br>12 (44,4)<br>*(p = 0,409)*	99 (42,7)<br>10 (37,0)<br>*(p = 0,263)*
**Insurance status**	Statutory health insurance<br>Private health insurance	112 (62,6)<br>60 (75,0)<br>*(p = 0,065)*	117 (65,4)<br>57 (71,3)<br>*(p = 0,133)*	109 (60,6)<br>53 (66,3)<br>*(p = 0,168)*	99 (55,0)<br>59 (72,8)<br>***(p = 0,012)***	102 (56,7)<br>60 (75,0)<br>***(p = 0,017)***	100 (55,6)<br>57 (71,3)<br>***(p = 0,049)***	99 (55,3)<br>48 (59,3)<br>*(p = 0,592)*	75 (41,9)<br>35 (43,8)<br>*(p = 0,669)*
**Knowledge of the definition of eHealth**	No<br>Yes	116 (59,2)<br>57 (86,4)<br>***(p < 0,001)***	120 (61,2)<br>54 (81,8)<br>***(p = 0,001)***	110 (55,8)<br>53 (80,3)<br>***(p < 0,001)***	109 (55,1)<br>51 (77,3)<br>***(p = 0,002)***	111 (56,3)<br>53 (80,3)<br>***(p < 0,001)***	108 (54,8)<br>51 (77,3)<br>***(p = 0,003)***	96 (49,0)<br>53 (79,1)<br>***(p < 0,001)***	70 (35,7)<br>41 (62,1)<br>***(p < 0,001)***
**Medication intake**	≤ 5 different medication/day<br>≥ 6 different medication/day	108 (67,1)<br>64 (65,3)<br>*(p = 0,930)*	105 (65,2)<br>67 (68,4)<br>*(p = 0,543)*	99 (61,1)<br>62 (63,3)<br>*(p = 0,939)*	98 (60,1)<br>61 (62,2)<br>*(p = 0,936)*	105 (64,8)<br>58 (59,2)<br>*(p = 0,376)*	98 (60,5)<br>60 (61,2)<br>*(p = 0,940)*	95 (58,6)<br>54 (55,1)<br>*(p = 0,817)*	68 (42,2)<br>42 (42,9)<br>*(p = 0,100)*
**Participation before COVID-19**	Yes<br>No	47 (64,4)<br>126 (66,7)<br>(p = 0,215)	46 (63,0)<br>128 (67,7)<br>(p = 0,164)	41 (56,2)<br>122 (64,2)<br>(p = 0,167)	43 (58,9)<br>117 (61,3)<br>(p = 0,295)	44 (60,3)<br>120 (63,2)<br>(p = 0,105)	41 (56,2)<br>118 (62,1)<br>(p = 0,099)	35 (47,9)<br>115 (60,0)<br>(p = 0,210)	26 (35,6)<br>85 (45,0)<br>(p = 0,302)
**Reasons for medical consultation**	Active therapy<br>Follow up care	127 (63,1)<br>34 (82,9)<br>(p = 0,040)	139 (64,1)<br>33 (80,5)<br>(p = 0,122)	132 (60,6)<br>29 (70,7)<br>(p = 0,436)	128 (58,4)<br>30 (73,2)<br>(p = 0,189)	131 (60,1)<br>31 (75,6)<br>(p = 0,162)	128 (58,7)<br>29 (70,7)<br>(p = 0,349)	117 (53,7)<br>30 (73,2)<br>(p = 0,058)	88 (40,6)<br>21 (51,2)<br>(p = 0,447)
**Type of cancer**	Solid<br>Hematological	80 (64,5)<br>82 (71,9)<br>(p = 0,312)	84 (67,7)<br>78 (68,4)<br>(p = 0,845)	79 (63,7)<br>71 (62,3)<br>(p = 0,940)	73 (58,4)<br>77 (67,5)<br>(p = 0,272)	75 (60,5)<br>78 (68,4)<br>(p = 0,325)	71 (57,3)<br>76 (66,7)<br>(p = 0,254)	70 (56,0)<br>68 (60,2)<br>(p = 0,610)	53 (42,7)<br>50 (43,9)<br>(p = 0,952)

S8 Table. Cancer patients attitude towards eHealth.
