# Supplementary material for: The potential of eHealth for cancer patients–does COVID-19 pandemic change the attitude towards use of telemedicine services?
Source: PLoS One. 2023 Feb 10;18(2):e0280723. doi: 10.1371/journal.pone.0280723 (PMC9917238; doi:10.1371/journal.pone.0280723)
Supplement: S9 Table — (PDF) [file pone.0280723.s009.pdf]

		% Patients have concerns about data security in online doctor-patient contact				
		**Via e-mail**	**Via health app**	**Via messenger**	**Via video consultation**	**Data security concerns regarding online appointment scheduling**
		Total:<br>N = 268<br>Yes: n = 85<br>No: n = 148<br>I.d.k.: n = 35	Total:<br>N = 267<br>Yes n = 97<br>No: n = 118<br>I.d.k.: n = 52	Total:<br>N = 267<br>Yes: n = 126<br>No: n = 95<br>I.d.k.: n = 46	Total:<br>N = 260<br>Yes: n = 79<br>No: n = 124<br>I.d.k.: n = 57	Total:<br>N = 268<br>Yes n = 51<br>No: n = 183<br>I.d.k.: n = 34
**Gender**	Female<br>Male	41 (32,5)<br>44 (31,7)<br>*(p = 0,988)*	44 (35,2)<br>52 (37,4)<br>*(p = 0,538)*	52 (41,6)<br>73 (52,5)<br>*(p = 0,109)*	32 (26,0)<br>46 (33,8)<br>*(p = 0,197)*	26 (20,6)<br>25 (18,0)<br>*(p = 0,795)*
**Age**	≤ 54<br>≥ 55	25 (37,3)<br>59 (30,1)<br>***(p = 0,005)***	32 (47,8)<br>63 (32,3)<br>***(p = 0,001)***	43 (64,2)<br>81 (41,5)<br>***(p < 0,001)***	22 (33,3)<br>55 (28,8)<br>***(p = 0,011)***	15 (22,4)<br>34 (17,3)<br>*(p = 0,166)*
**Community size (Inhabitants)**	>= 30.000<br>> 30.000	43 (31,6)<br>40 (32,8)<br>*(p = 0,029)*	40 (29,4)<br>54 (44,6)<br>***(p = 0,001)***	56 (41,2)<br>66 (54,5)<br>***(p = 0,05)***	34 (26,0)<br>42 (34,7)<br>***(p = 0,004)***	20 (21,3)<br>20 (16,4)<br>*(p = 0,029)*
**Proximity to university hospital**	≤ 20 km<br>≥ 21 km	44 (33,3)<br>41 (31,1)<br>(p = 0,184)	52 (39,7)<br>44 (33,3)<br>(p = 0,091)	62 (47,3)<br>63 (47,7)<br>(p = 0,343)	36 (27,7)<br>42 (32,8)<br>(p = 0,209)	32 (24,2)<br>19 (14,4)<br>(p = 0,049)
**Travel time to hospital**	≤ 30 min<br>≥ 31 min	40 (28,6)<br>44 (36,1)<br>(p = 0,064)	54 (38,6)<br>42 (34,4)<br>(p = 0,051)	66 (47,1)<br>59 (48,4)<br>(p = 0,105)	39 (28,7)<br>39 (32,2)<br>(p = 0,240)	30 (21,4)<br>20 (16,4)<br>(p = 0,082)
**Educational level**	Low<br>Middle + high	26 (32,5)<br>59 (32,8)<br>***(p = 0,008)***	22 (27,8)<br>73 (40,6)<br>*(p = 0,017)*	30 (38,0)<br>94 (52,2)<br>*(p = 0,077)*	24 (30,8)<br>54 (30,5)<br>*(p = 0,452)*	16 (20,0)<br>34 (18,9)<br>***(p = 0,043)***
**Occupational level**	Low<br>Middle + high	9 (34,6)<br>76 (32,3)<br>***(p = 0,011)***	6 (23,1)<br>89 (38,0)<br>***(p = 0,027)***	9 (34,6)<br>115 (49,1)<br>***(p = 0,116)***	7 (29,2)<br>71 (30,6)<br>***(p = 0,685)***	8 (30,8)<br>43 (18,3)<br>(p = 0,087)
**Employed**	No<br>Yes	68 (34,9)<br>17 (25,4)<br>***(p = 0,018)***	71 (36,6)<br>25 (37,3)<br>*(p = 0,350)*	90 (46,4)<br>35 (52,2)<br>*(p = 0,437)*	64 (33,3)<br>14 (21,5)<br>***(p = 0,014)***	36 (18,5)<br>15 (22,4)<br>*(p = 0,109)*
**Full time or part time job**	≤ 50%<br>> 50 %	6 (24,0)<br>13 (29,5)<br>*(p = 0,880)*	9 (36,0)<br>17 (38,6)<br>*(p = 0,844)*	11 (44,0)<br>24 (54,5)<br>*(p = 0,596)*	3 (12,5)<br>11 (25,6)<br>*(p = 0,284)*	7 (28,0)<br>9 (20,5)<br>*(p = 0,742)*
**Frequency of medical consultation in the last year**	≤ 5 times<br>> 5 times	14 (32,6)<br>70 (32,0)<br>***(p = 0,071)***	13 (30,2)<br>84 (38,4)<br>*(p = 0,411)*	18 (41,9)<br>108 (49,3)<br>*(p = 0,204)*	11 (28,2)<br>68 (31,3)<br>*(p = 0,625)*	10 (23,3)<br>40 (18,3)<br>*(p = 0,034)*
**Missed appointments in the past**	No<br>Yes	77 (32,4)<br>8 (29,6)<br>*(p = 0,928)*	86 (36,3)<br>11 (40,7)<br>*(p = 0,770)*	110 (46,4)<br>15 (55,6)<br>*(p = 0,322)*	68 (29,4)<br>10 (38,5)<br>*(p = 0,568)*	42 (17,6)<br>7 (25,9)<br>*(p = 0,502)*
**Insurance status**	Statutory health insurance<br>Private health insurance	61 (33,7)<br>24 (28,9)<br>*(p = 0,492)*	63 (35,0)<br>33 (39,8)<br>*(p = 0,586)*	83 (46,1)<br>42 (50,6)<br>*(p = 0,794)*	54 (30,2)<br>24 (30,4)<br>*(p = 0,170)*	38 (21,0)<br>13 (15,7)<br>***(p = 0,044)***
**Knowledge of the definition of eHealth**	No<br>Yes	60 (29,9)<br>25 (37,3)<br>***(p = 0,001)***	67 (33,5)<br>30 (44,8)<br>***(p = 0,002)***	87 (43,5)<br>39 (58,2)<br>***(p = 0,001)***	57 (29,4)<br>22 (33,3)<br>***(p = 0,013)***	38 (18,9)<br>13 (19,4)<br>***(p = 0,001)***
**Medication intake**	≤ 5 different medication/day<br>≥ 6 different medication/day	53 (32,1)<br>32 (32,0)<br>*(p = 0,054)*	57 (34,8)<br>38 (38,0)<br>***(p = 0,014)***	78 (47,6)<br>46 (46,0)<br>***(p = 0,045)***	43 (26,9)<br>35 (36,1)<br>***(p = 0,012)***	28 (17,0)<br>23 (23,0)<br>*(p = 0,229)*
**Participation before COVID-19**	Yes<br>No	25 (34,2)<br>60 (30,8)<br>(p = 0,812)	28 (38,4)<br>69 (35,6)<br>(p = 0,822)	32 (43,8)<br>94 (48,5)<br>(p = 0,772)	21 (28,8)<br>58 (31,0)<br>(p = 0,916)	9 (12,3)<br>42 (21,5)<br>(p = 0,232)
**Reasons for medical consultation**	Active therapy<br>Follow up care	72 (32,6)<br>13 (31,0)<br>(p = 0,941)	88 (40,0)<br>9 (21,4)<br>(p = 0,073)	111 (50,5)<br>15 (35,7)<br>(p = 0,194)	71 (32,7)<br>8 (20,0)<br>(p = 0,169)	42 (19,0)<br>9 (21,4)<br>(p = 0,936)
**Type of cancer**	Solid<br>Hematological	42 (33,6)<br>37 (32,5)<br>(p = 0,059)	49 (38,3)<br>41 (36,0)<br>(p = 0,312)	58 (45,3)<br>61 (53,5)<br>(p = 0,348)	38 (30,4)<br>36 (31,9)<br>(p = 0,867)	22 (17,2)<br>25 (21,9)<br>(p = 0,070)

S9 Table. Data security concerns.
